# Engineering a Programmed Death-Ligand 1-Targeting Monobody Via Directed Evolution for SynNotch-Gated Cell Therapy

**DOI:** 10.1021/acsnano.4c01597

**Published:** 2024-03-08

**Authors:** Linshan Zhu, Chi-Wei Man, Reed E.S. Harrison, Zhuohang Wu, Praopim Limsakul, Qin Peng, Matthew Hashimoto, Anthony P. Mamaril, Hongquan Xu, Longwei Liu, Yingxiao Wang

**Affiliations:** † Department of Bioengineering & Institute of Engineering in Medicine, University of California, San Diego, La Jolla, California 92093, United States; ‡ Alfred E. Mann Department of Biomedical Engineering, University of Southern California, Los Angeles, California 90089, United States; § Department of Chemistry and Biochemistry, University of California, San Diego, La Jolla, California, 92093 United States; ∥ Institute of Systems and Physical Biology, Shenzhen Bay Laboratory, Shenzhen 518132, P.R. China; ⊥ Division of Physical Science, Faculty of Science, Prince of Songkla University, Hat Yai 90110, Songkhla, Thailand; # Center of Excellence for Trace Analysis and Biosensor, Prince of Songkla University, Hat Yai 90110, Songkhla, Thailand; ∇ Department of Statistics, University of California, Los Angeles, California 90095, United States

**Keywords:** PD-L1, Monobody, Yeast surface display, Directed evolution, CAR T cell therapy, SynNotch

## Abstract

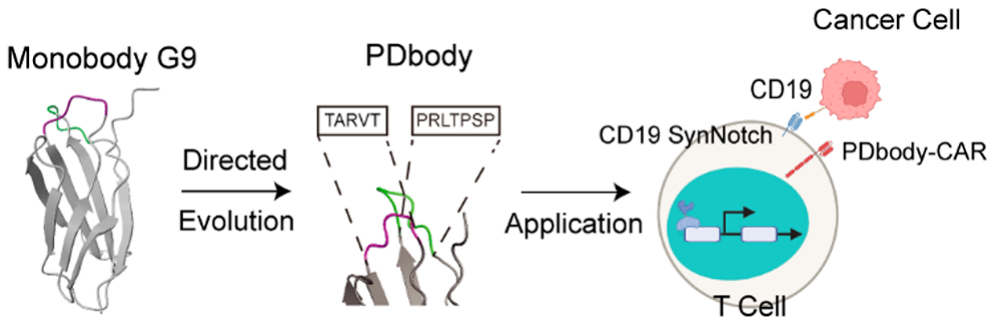

Programmed death-ligand 1 (PD-L1) is a promising target for cancer immunotherapy due to its ability to inhibit T cell activation; however, its expression on various noncancer cells may cause on-target off-tumor toxicity when designing PD-L1-targeting Chimeric Antigen Receptor (CAR) T cell therapies. Combining rational design and directed evolution of the human fibronectin-derived monobody scaffold, “PDbody” was engineered to bind to PD-L1 with a preference for a slightly lower pH, which is typical in the tumor microenvironment. PDbody was further utilized as a CAR to target the PD-L1-expressing triple negative MDA-MB-231 breast cancer cell line. To mitigate on-target off-tumor toxicity associated with targeting PD-L1, a Cluster of Differentiation 19 (CD19)-recognizing SynNotch IF THEN gate was integrated into the system. This CD19-SynNotch PDbody-CAR system was then expressed in primary human T cells to target CD19-expressing MDA-MB-231 cancer cells. These CD19-SynNotch PDbody-CAR T cells demonstrated both specificity and efficacy *in vitro*, accurately eradicating cancer targets in cytotoxicity assays. Moreover, in an *in vivo* bilateral murine tumor model, they exhibited the capability to effectively restrain tumor growth. Overall, CD19-SynNotch PDbody-CAR T cells represent a distinct development over previously published designs due to their increased efficacy, proliferative capability, and mitigation of off-tumor toxicity for solid tumor treatment.

Chimeric Antigen Receptor T (CAR T) cell therapy is a revolutionary treatment option for cancer therapy.^1,2^ CAR consists of an extracellular antigen recognition domain (usually a Single-Chain Fragment Variable (scFv)), a hinge, a transmembrane domain, and intracellular costimulatory and signaling domains.^3,4^ Following the recognition of a specified antigen on the cancer cell surface, CAR T cells induce cytotoxicity by triggering endogenous T cell activation pathways.^5−8^ CAR T cell therapy has demonstrated outstanding efficacy in treating hematological cancers, but solid tumors remain a challenge to treat.^9^ Multiple factors contribute to this challenge, including the lack of tumor-specific antigens as well as the local immunosuppressive tumor microenvironment.^10,11^

Immune checkpoint inhibition by PD-L1 provides negative regulatory feedback and suppresses T cell activation.^12,13^ This negative regulatory function creates a survival advantage for cancer cells that upregulate PD-L1. Indeed, cancer cells with upregulated PD-L1 levels are found in many cancer types, including nonsmall cell lung cancer (NSCLC), head and neck squamous cell carcinoma (HNSCC), Hodgkin’s lymphoma, and renal cell carcinoma.^13^ Accordingly, immune checkpoint blockade (ICB) of the PD-1/PD-L1 axis is an effective treatment for a number of cancers.^14,15^ ICB treatment usually involves the application of a PD-1 or PD-L1 blocking antibody in combination with one or more other treatments, a strategy referred to as combination therapy. The success of these strategies demonstrates the importance of the PD-1/PD-L1 axis in cancer therapy.^16−18^

Thus, targeting PD-L1 as a CAR T cell antigen is an exciting strategy. By targeting cancer cells overexpressing PD-L1, CAR T cells can not only be guided to attack cancer cells but also neutralize the immunosuppressive PD-1/PD-L1 axis and mitigate T cell exhaustion. Despite the promise of this approach, it is particularly risky because of potential on-target off-tumor effects. In fact, in addition to its upregulation in cancer cells, PD-L1 is expressed in various other cell types, including but not limited to T cells, B cells, dendritic cells, macrophages, and vascular endothelial cells.^13^ Off-tumor CAR T cell attack can lead to cytokine release syndrome and, in the worst-case scenario, even death.^19−23^ For this reason, there is currently no FDA-approved CAR T cell developed to target PD-L1. Design strategies are particularly needed to avoid off-target toxicity when targeting PD-L1.^24^ To avoid nonspecific toxicity, two strategies were employed in our study: (1) the design of a CAR based on a PD-L1-recognizing monobody CAR with stronger affinity at a relatively lower pH typical in the tumor microenvironment^25^ and (2) the integration with SynNotch recognizing a clinically validated tumor-associated antigen (TAA) CD19 introduced into target cancer cells to form an IF THEN gate with PD-L1 for high precision control.

The monobody is a low molecular weight (10–15 kDa), single domain Ig-like protein scaffold derived from the 10th repeat of human fibronectin III^26−28^ (Figure 1A). Engineered to serve as a CAR receptor, the monobody provides a number of advantages compared to the standard scFv; (1) its small size makes it easier to package into lentiviruses,^29^ (2) its single domain nature prevents domain swapping which should reduce the risk of tonic signaling,^30^ (3) its human origin reduces the risk of immunogenicity,^31,32^ and (4) the monobody is more straightforward to engineer with three loops that are well-studied and most frequently engineered, compared to the six binding loops and linker region of scFvs. These features of the monobody should avoid the aggregation tendencies and inefficient folding typical of scFvs^33,34^ as well as the chronic activation and tonic signaling of a reported PD-L1 nanobody.^24^ For this study, a combination of rational design and directed evolution was used to engineer the BC and FG loops of the monobody to bind to PD-L1. Studies have shown that CARs with moderate affinities are better suited to distinguish low versus high density of antigens on target cells and, hence, are designed in some clinical treatments to specifically avoid off-tumor toxicity against healthy tissues/cells expressing low levels of target antigen.^35−39^ Thus, the monobody CAR in this report was engineered with moderate binding affinity and potentially could specifically target cancer cells with high PD-L1 density which tend to resist drug treatment^40^ while sparing bystander cells expressing low levels of PD-L1.

To further address the issues associated with the ubiquitous expression of PD-L1 in the body, an IF THEN gate control SynNotch was added to the PD-L1-targeting system to provide localized targeting specificity.^41−43^ SynNotch has already demonstrated a variety of uses in immunotherapy.^44−48^ In our work, a clinically validated CD19 antigen was introduced to express on a subpopulation of MDA-MB-231 breast cancer cells, which served as “training centers” to engage SynNotch and induce PDbody-CAR production in engineered T cells. PDbody-CAR targeted PD-L1, which is universally expressed on MDA-MB-231 cells, to eradicate the entire tumor population. Without the introduced CD19 SynNotch ligand, CAR was not produced to target PD-L1, demonstrating an added layer of safety against on-target off-tumor toxicity. As such, our CD19-SynNotch-gated CAR with the PD-L1 targeting monobody provides a safer method to target cancer cells with high PD-L1 expression.

## Results and Discussion

### Monobody Scaffold Binds PD-L1 in Low pH

**Figure 1:**
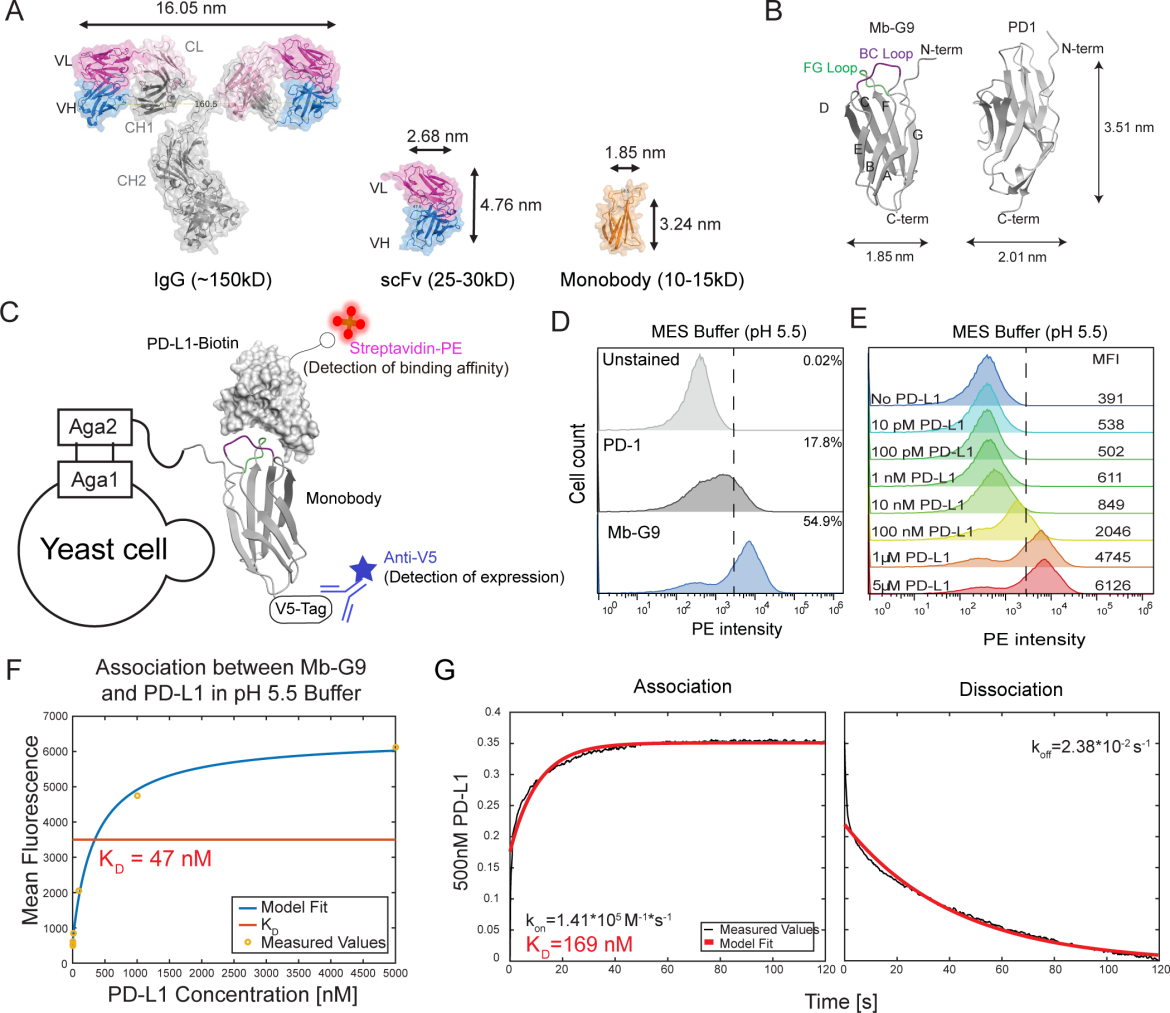
**Characterization of Mb-G9 binding affinity to PD-L1.** (A) Size comparison among antibody, scFv, and monobody. Specifically, the nanoscale monobody is advantageous due to its small size, stability, and ease of engineering for high specificity and affinity for its targets. (B) Crystal structure of monobody (ID: 1TTG) with loops and beta strands labeled. BC loop is shown in magenta, and the FG loop is in green. The structure illustration of PD-1 (amino acids 30–147) is predicted by AlphaFold. (C) Schematic illustration of the yeast staining principle. The V5 tag is used to verify the expression of monobody on the yeast surface. Biotinylated PD-L1 is added to assess the binding affinity of the monobody, which is then detected by the secondary staining of Streptavidin-PE. (D) PD-L1 binding of Mb-G9 in MES buffer (pH 5.5). PD-L1 binding of Mb-G9 and WT PD-1 are shown in blue and dark gray, respectively. Unstained cells are shown in light gray. The positive rates of PE-stained cells of different groups have been shown. (E) PD-L1 titration of Mb-G9. Biotinylated PD-L1 at varying concentrations was incubated to bind to yeast-displayed Mb-G9 and stained with streptavidin-PE. The mean fluorescent intensity (MFI) of PE was illustrated in the figures. (F) Mean fluorescence intensity values from (E) were plotted, and nonlinear least-squares regression was fit to the data points to calculate a *K*_D_ of 47 nM. (G) BLI measurement of PD-L1 binding affinity for purified Mb-G9 in MES buffer. Based on the kinetics data obtained, a *K*_D_ value of 169 nM was calculated.

The G9 monobody (Mb-G9) was originally engineered to bind to the SH3 domain of Fyn tyrosine protein kinase.^49^ As a member of the immunoglobulin-like domain family, its secondary structure is similar to that of human Programmed Death Receptor-1 (PD-1)^50^ (Figure 1B). Thus, it was chosen as a starting scaffold for further engineering to increase its affinity toward PD-L1. We initially employed yeast display to evaluate the binding affinity of Mb-G9 toward PD-L1, using Programmed cell death protein 1 (PD-1), the natural binding partner of PD-L1, as a positive control. To compare the affinity between Mb-G9 and PD-1, individual yeast clones are induced for surface expression and incubated with 5 μM biotinylated PD-L1 (PD-L1 BTN) (Supporting Information Figure 1) for 30 min at 25 °C to reach equilibrium. A secondary streptavidin-phycoerythrin (SA-PE) was used to stain the cells at 4 °C for 30 min. The PE high percentage or the mean fluorescence intensity of PE was used as a parameter to determine the binding affinity (Figure 1C). Surprisingly, PD-1 showed no apparent binding to PD-L1 when stained in phosphate-buffered saline (PBS) buffer (Supporting Information Figure 2), suggesting a weak physiological binding affinity of PD-1 toward PD-L1 as previously reported.^51^ PD-L1 binding of PD-1 was only observed after changing the buffer to pH 5.5 2-(*N*-morpholino)ethanesulfonic acid (MES) buffer (Figure 1D), which was earlier reported to boost PD-L1 binding.^52^ Mb-G9 also showed PD-L1 binding in the MES buffer at slightly higher levels than that of PD-1.

Further staining experiments showed that increased binding was significantly affected by the pH of the buffers. Indeed, when MES buffer was titrated from pH 5.5 to 6.9, PD-L1 binding of Mb-G9 decreased. Likewise, when PBS buffer was titrated from pH 7.4 to pH 6.0, PD-L1 binding slightly increased (Supporting Information Figure 3A,B). Interestingly, PD-L1 binding toward Mb-G9 in pH 6.9 MES buffer was stronger than binding in pH 6.0 PBS buffer, suggesting that buffer composition also plays an important role in regulating PD-L1 binding. Because PD-L1 binding was weak in PBS buffers, initial comparisons of binding between monobody variants were performed in a pH 5.5 MES buffer. Library screening was thereafter performed in pH 6.5 MES buffers to better mimic physiological conditions.

To measure the affinity of Mb-G9, we used yeast surface display. Yeast cells were cultured and induced to express the Mb-G9 on the cell surface. 1 × 10^7^ yeast cells were mixed with PD-L1 BTN at range of concentrations from 10 pM to 5 μM, which is around the expected *K*_D_, and allowed to reach equilibrium at 25 °C for 45 min. Cells were then further stained with SA-PE on ice for 15 min and examined by flow cytometry (Figure 1C). As can be seen in the flow cytometry graph, the magnitude of binding was correlated with the added amount of PD-L1. Mean fluorescence intensities were extracted from flow cytometry plots, and nonlinear squares regression^53^ was used to calculate the dissociation constant *K*_D_ of Mb-G9 to be 47 nM in pH 5.5 MES buffer (Figure 1E,F). To crosscheck PD-L1 binding with a different method, standard biolayer interferometry (BLI) was used to directly measure the binding affinity between purified PD-L1 and Mb-G9. *K*_D_, *k*_on_, and *k*_off_ values were determined from kinetics measurements using nonlinear squares regression. At 500 nM PD-L1, the *K*_D_ value of purified biotinylated Mb-G9 was measured to be 169 nM, which is in the similar range of the Mb-G9 *K*_D_ measured by yeast display and flow cytometry (Figure 1G). pH 5.5 MES buffer was used here for both flow cytometry and BLI analysis.

Before starting the process of directed evolution, more information was desired about where Mb-G9 binds to PD-L1 and which amino acid residues might be key in its binding. In an inhibition assay, PD-1 was expressed via yeast surface display, and PD-L1 binding was examined with titrated Mb-G9. The group with added Mb-G9 showed less binding to PD-L1 (Supporting Information Figure 3C). This suggests that Mb-G9 may compete against PD-1 in binding to PD-L1 via the same binding pocket and could thus act as a competitive inhibitor. To further probe which monobody residues may play an important role in PD-L1 binding, the BC and FG loop regions were mutated to see whether PD-L1 binding would be significantly affected following an earlier publication.^54^ Results suggested that binding did not significantly change in the MES buffer (Supporting Information Figure 3D). Thus, a more systematic and high-throughput method of protein engineering was required to further improve the monobody affinity toward PD-L1.

### Affinity Maturation of Monobody toward PD-L1

**Figure 2:**
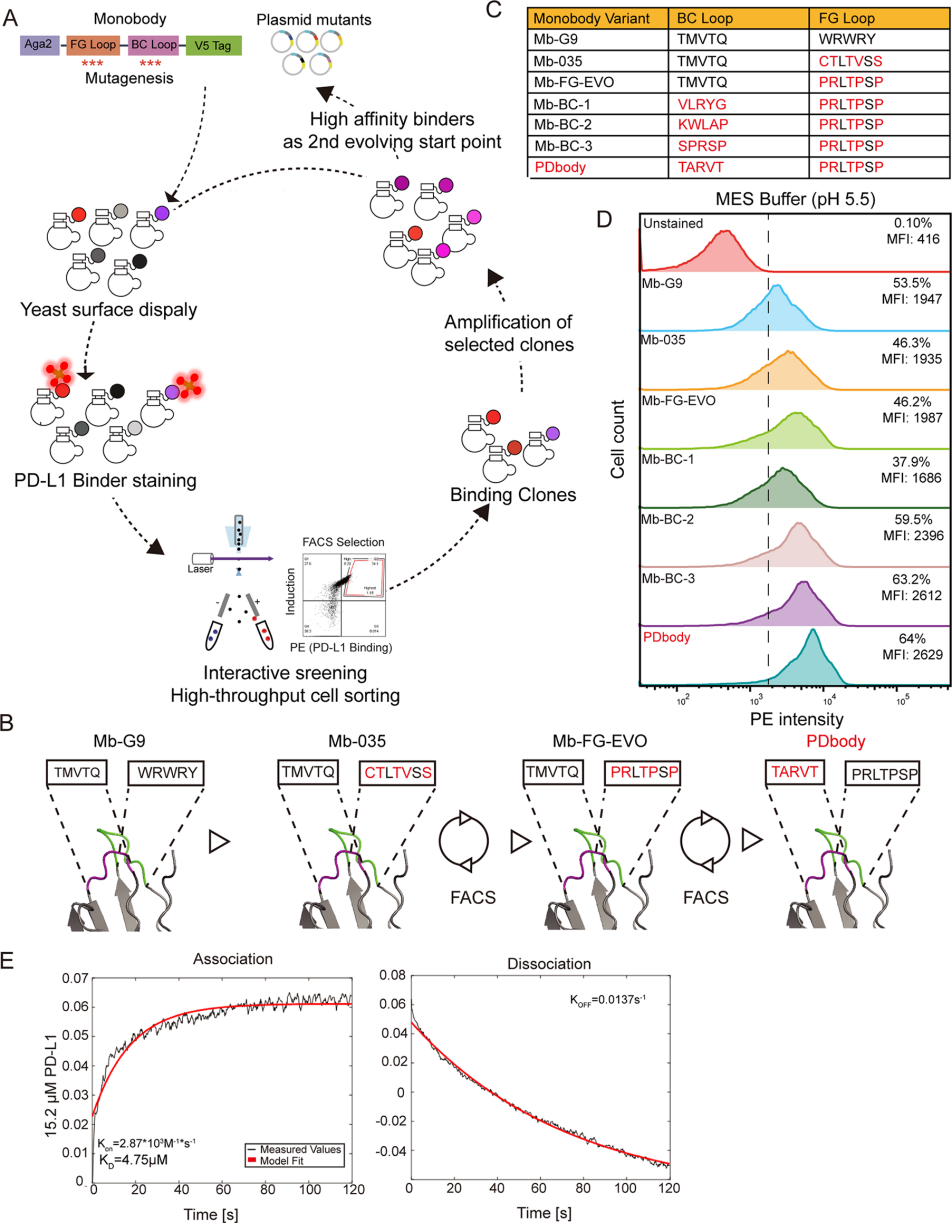
**Directed evolution of the high affinity PD-L1 binding monobody.** (A) Protein engineering workflow based on directed evolution and yeast surface display to select high affinity PD-L1 binders. Yeast libraries on FG and BC loops are generated by site-saturated mutagenesis and were induced to express on the yeast surface. Biotinylated PD-L1 and streptavidin-PE were then used to quantify PD-L1 binding levels. To enrich for higher affinity PD-L1 binders, FACS was used to sort out the top 1% of library populations. Sorted binding clones are then further amplified and enter the second round of screening. (B) Monobody variants are sequentially obtained through a combination of rational design and directed evolution, and their BC and FG loop sequences are shown, respectively, in magenta and green. Mb-G9 represents the starting scaffold. Mb-035 is obtained after grafting the KN-035 peptide into the FG loop. Rational design is used to guide the selection of amino acid residues to mutate for the FG loop library which resulted in the generation of Mb-FG-EVO. Ultimately, PDbody is obtained by creating a BC loop library of Mb-FG-EVO and undergoing another iteration of library screening. (C) Table of monobody variants and their amino acid sequences obtained through library screening. (D) The binding affinity of monobody variants to PD-L1. Yeast-displayed monobody variants were stained with 5 μM biotinylated PD-L1 and streptavidin-PE in pH 5.5 MES buffer. (E) BLI measurement of PD-L1 binding for PDbody in pH 7.4 PBS buffer. Based on the kinetics data obtained, a *K*_D_ value of 4.75 μM was calculated.

To systematically improve the binding affinity of the monobody, we employed a directed evolution approach involving multiple rounds of random mutagenesis, affinity screening using Fluorescence Activated Cell Sorting (FACS), and variant identification and verification (Figure 2A). We improved the initial binding affinity of Mb-G9 by grafting a PD-L1 binding peptide sequence into the FG loop (Mb-035). A site-saturated FG-loop library guided by rational design was then created on top of Mb-035. The clone that resulted from the FG-loop library screening was dubbed Mb-FG-EVO. Finally, a BC loop library was generated on top of Mb-FG-EVO, which was screened through another round of directed evolution, and the PDbody sequence was determined (Figure 2B).

Since the success of optimizing a protein binder via directed evolution is dependent on choosing a good starting point,^55^ the KN-035 CDR loop^56^ was grafted into the FG loop region of the monobody to create Mb-035 to increase the basal PD-L1 binding before directed evolution. The KN-035 loop was originally part of a PD-L1-binding nanobody, but the monobody is advantageous to the nanobody because the monobody is human-derived and, hence, potentially less immunogenic. In addition, CAR engineered by using PD-L1-binding nanobody has shown severe chronic activation and tonic signaling.^24^ Yeast staining results showed no significant difference in PD-L1 binding between Mb-035 and Mb-G9 in physiological pH PBS buffer (Supporting Information Figure 4A), but staining in pH 5.5 MES buffer showed a slightly higher level of PD-L1 binding in Mb-035 (Supporting Information Figure 4B).

Specifically, to identify which residues of the KN-035 loop to mutate for directed evolution, molecular dynamics simulations were performed between Mb-035 and PD-L1. Based on molecular dynamics simulations, residues were deemed suitable targets for mutation and optimization if they spent a long time in close proximity to PD-L1 but not directly interacted with PD-L1 (Supporting Information Figure 4C). Accordingly, residues targeted for optimization are shown in cyan, with less optimizable residues shown in magenta (Supporting Information Figure 4D,E). Thus, site-saturated mutagenesis was performed on identified target residues C82, T83, V85, T86, and T88. Monobody libraries generated using site-saturated mutagenesis were displayed on the yeast surface and then stained by PD-L1 BTN, which was then detected by the dye SA-PE. Clones with the brightest PE signals were sorted via FACS (Figure 2A). After site-saturated mutagenesis and directed evolution screening of the FG loop library, the Mb-FG-EVO was obtained. The FG loop sequence of this improved variant is PRLTPSP, which is significantly different from the CTLTVSS of the original Mb-035 scaffold. Consistently, PD-L1 staining of monobody variant Mb-FG-EVO showed a clear improvement in PD-L1 binding compared to previous variants (Supporting Information Figure 4F). While improvement in PD-L1 binding was noticeable, overall binding was still relatively moderate.

To further improve the monobody affinity, site-saturated mutagenesis was performed on the BC loop of Mb-FG-EVO (Residues 26–30). Screening was performed using MES buffer at pH 6.5 to perform robust staining of clones. After four rounds of FACS screening, a number of higher-affinity mutants were isolated and gathered (Figure 2C). Four promising candidates with BC loop sequences VLRYG, KWLAP, SPRSP, and TARVT were tested. PD-L1 binding in the MES buffer showed that the BC loop sequence TARVT (PDbody) displayed stronger PD-L1 binding (Figure 2D) than those of other monobody variants at similar expression levels (Supporting Information Figure 5A). The binding affinity of purified PDbody (Supporting Information Figure 5B) was further measured by BLI and quantified to be 4.75 μM (Figure 2E) in PBS buffer, which is stronger than the 8.2 μM *K*_D_ of wild-type PD-1.^57^ This measured binding affinity also suggests a significant improvement to that of Mb-G9, which was previously undetectable by BLI in PBS. In conclusion, through interactive directed evolution and optimization of FG-loop and BC-loop, we have identified medium-affinity binders of PD-L1, namely, PDbody.

### Monobody Variants as CAR Receptors

**Figure 3:**
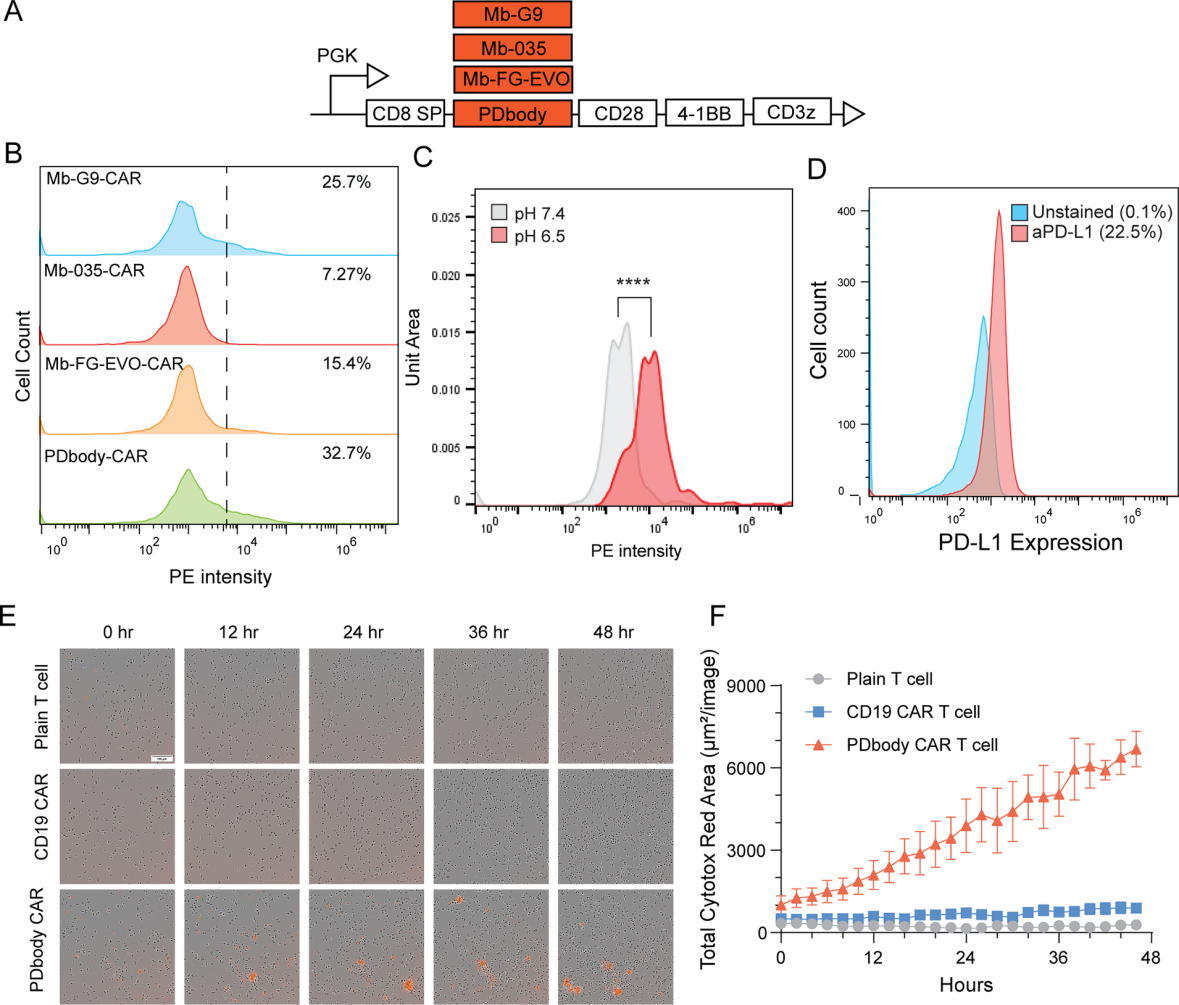
**Characterization of PDbody CAR T cells.** (A) Genetic cassettes of the monobody CAR constructs. Various monobody variants are integrated as receptor domains in a third-generation CAR. (B) The binding of monobody CARs to PD-L1. 1 μM biotinylated PD-L1 and streptavidin-PE were used to stain Jurkat-displayed monobody CARs in pH 7.4 PBS buffer. (C) Binding affinity of PDbody-CAR cells to PD-L1, assessed in buffers of two pH levels. Equal numbers of PDbody-CAR cells were stained with 1.675 μM PD-L1 and streptavidin-PE and washed in either pH 7.4 PBS or pH 6.5 PBS (30 mL of PBS acidified with 14 μL of 10 M HCl). A one-sided Wilcoxon rank-sum test confirms that the median of PD-L1 binding of PDbody-CAR at pH 7.4 is significantly less than that of PDbody-CAR at pH 6.5 with a *p*-value < 2.2 × 10^–16^ (medians: 2075 vs 9792). (D) Measurement of PD-L1 expression in primary T cells. T cells were stained with anti-PD-L1 PE antibody to verify PD-L1 expression levels. (E) Incucyte images of plain, CD19 CAR, and PDbody CAR T cells cultured with Cytotox Red dye. The Cytotox Red dye is used to stain the dead cells. (F) Quantification of the Cytotox Red dye signal over the period of 48 h culture.

To be used for immunotherapy, monobody variants were tested as cancer-recognition motifs in CAR receptors. Driven by Phosphoglycerate Kinase 1 Promoter (PGK) promoter, monobody variants were fused to CD28 transmembrane domain and CD28 and 4–1BB costimulatory domains for the generation of CARs (Figure 3A). These monobody CARs were then expressed in Jurkat cells. To test for PD-L1 binding of monobody CARs at similar expression levels (Supporting Information Figure 6A), PD-L1 staining was performed on each of the monobody variants. Results showed that PD-L1 binding of PDbody was higher than any of Mb-G9, Mb-035, or Mb-FG-EVO (Figure 3B). PD-L1 binding by PDbody-CAR was detected at 100 nM and 1 μM PD-L1 concentrations, which is consistent with the previously measured binding affinity of PDbody (Supporting Information Figure 6B). Furthermore, an increase in PD-L1 binding was observed at lower pH, which should prove favorable and more specific for the acidic tumor microenvironment (Figure 3C). However, during the production of our PDbody CAR T cells, the T cells showed limited expansion and proliferation. We compared the exhaustion markers of CD19 CAR and PDbody CAR in primary human T cells 7 days after CAR transduction but did not observe significant T cell exhaustion (Supporting Information Figure 6C). Further investigation revealed that primary T cells inherently express PD-L1 on their surface (Figure 3D), a finding also confirmed in previously published studies.^58−60^ This expression may lead to the autotoxicity of the PDbody CAR-T cells. In fact, we observed self-killing of the PDbody CAR T cells under an incucyte system using the Cytotox red dye, which stains dead cells (Figure 3E,F). These results suggest that PDbody CAR T cells might not be suitable for large-scale production and have a high risk of on-target off-tumor effects against normal cells expressing PD-L1, including the PDbody CAR T cells themselves. Based on these findings, a safer and more selective CAR T cell design should be employed to mitigate potential on-target off-tumor toxicity.

### SynNotch-Gated PDbody-CAR In Vitro

**Figure 4:**
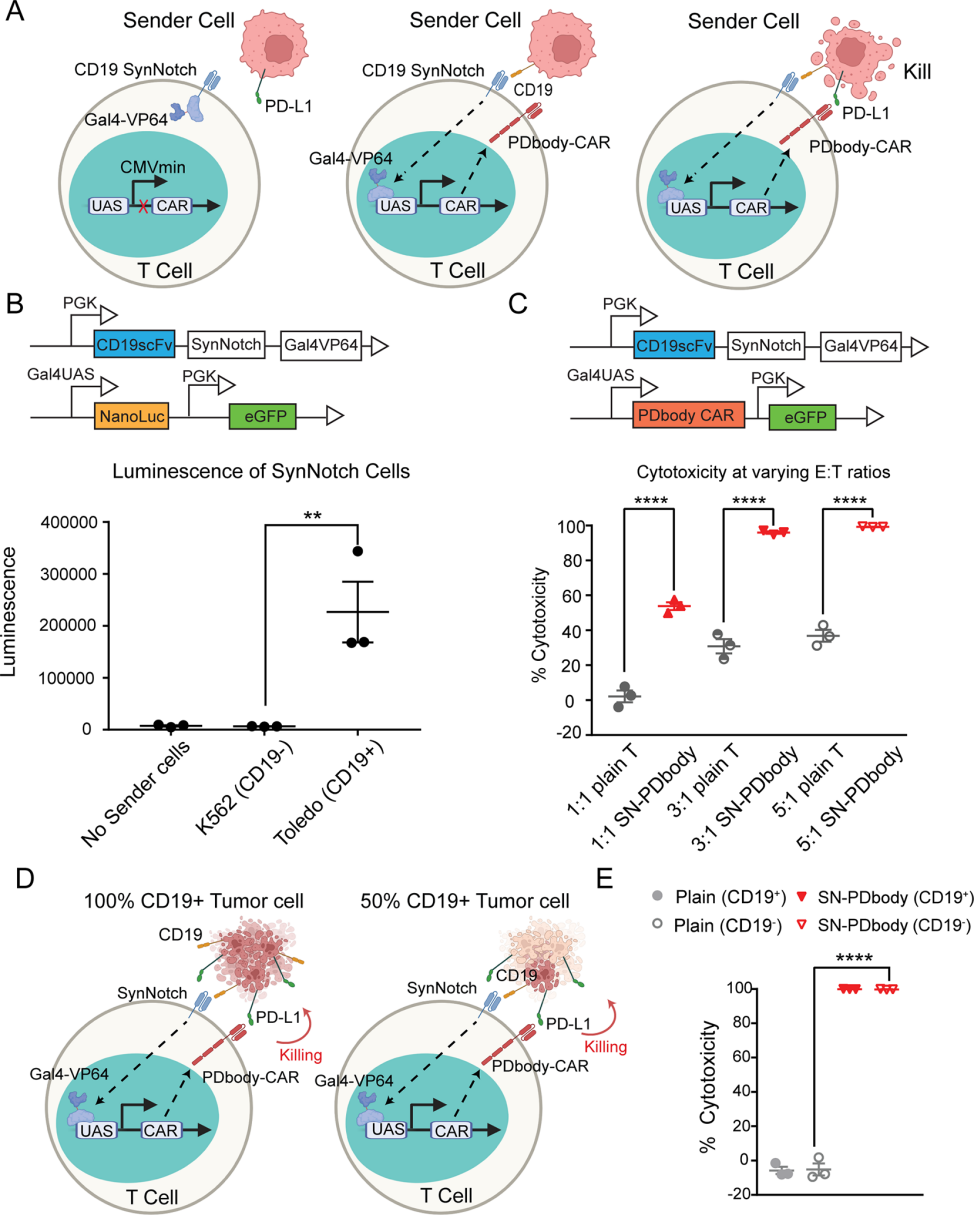
**PDbody can be integrated with the SynNotch system to eliminate PD-L1-expressing MDA-MB-231 cancer cells *in vitro*.** (A) Schematics of the IF THEN gate functionality of CD19-SynNotch PDbody-CAR. Sender cells lacking CD19 (sensing antigen) will not trigger PDbody-CAR expression and the subsequent tumor killing, even if PD-L1 (target antigen) is available (left). Cells with CD19 but without PD-L1 will induce the expression of PDbody-CAR but will not initiate T cell killing due to the absence of the target antigen (middle). Cancer cell destruction occurs only when both CD19 and PD-L1 are expressed (right). (B) Verification of CD19-SynNotch PDbody-CAR. αCD19 SynNotch receptor is used in combination with nanoluciferase in the reporter construct. Verification involves Toledo cells (CD19^+^) and K562 cells (CD19^−^). Nanoluciferase induction occurs only in the presence of the CD19 antigen. (C) Cytotoxicity of CD19-SynNotch PDbody-CAR T cells at varying E/T ratios. Untransduced plain T cells and CD19-SynNotch PDbody-CAR T cells were cocultured with 100% CD19^+^ MDA-MB-231 cells at 1:1, 3:1, and 5:1 E/T ratios. Cytotoxicity of plain and CD19-SynNotch PDbody-CAR T cells is shown in light gray and red, respectively. (D) Schematic illustrating the “training center” principle using CD19-SynNotch PDbody-CAR T cells. With 50% CD19^+^ cells, the SynNotch system is activated, inducing PDbody CAR expression. This enables the killing of nearby PD-L1 positive cells, including the remaining 50% CD19^–^ cancer cells. (E) Cytotoxicity of CD19-SynNotch PDbody-CARs. T cells were cocultured for 24 h in a 3:1 ratio with a 1:1 mixture of CD19^+^ and CD19^–^ MDA-MB-231 cells. Cytotoxicity of plain cells and CD19-SynNotch PDbody-CAR are shown in light gray and red, respectively.

As PD-L1 is expressed in a broad range of cell types and our PDbody CAR T cells showed autotoxicity, additional gating of PDbody should further improve the specificity of PDbody-based CAR T cell therapy. We reasoned that an IF THEN gate integrating SynNotch and PDbody-CAR should minimize off-tumor toxicity and increase the safety of the PDbody-CAR system as cytotoxicity will only occur when both a tumor-associated antigen (TAA) and PD-L1 are expressed on the target cell (Figure 4A). We first examined and verified the SynNotch system. A CD19 scFv SynNotch receptor and nanoluciferase reporter^61^ were transduced into Jurkat cells. These cells were either cultured alone (no sender cells) or cocultured with CD19-negative K562 cells or CD19-positive Toledo cells in a 1:1 ratio (Figure 4B). Luminescence measurements revealed that the SynNotch system was able to discern between CD19-negative and CD19-positive cells (Figure 4B).

Next, the nanoluciferase reporter was replaced by PDbody CAR and transduced into human primary T cells along with the CD19scFv SynNotch receptor (Figure 4C); FACS was used to sort and select T cells expressing both constructs (Supporting Information Figure 7A). MDA-MB-231 cells, a highly aggressive, invasive, and triple-negative breast cancer (TNBC) cell line lacking estrogen receptor (ER), progesterone receptor (PR), and human epidermal growth factor receptor 2 (HER2)^62,63^ but expressing high levels of endogenous PD-L1 (Supporting Information Figure 7B), were used as target cancer cells in luminescence-based killing assays. These MDA-MB-231 cells were transduced with a gene cassette encoding a truncated CD19 (ectodomain and transmembrane domain only) connecting to self-cleaving peptide P2A and firefly luciferase (Supporting Information Figure 7C) to create a CD19-positive cell line. MDA-MB-231 cells were also transduced with a myc-P2A-renilla luciferase construct to serve as a CD19-negative control. The luciferase signals were measured in triplicate to verify a proportional correlation with cell number (Supporting Information Figure 7D).

To test killing specificity, CD19-SynNotch PDbody-CAR T cells were first cocultured with CD19-negative MDA-MB-231 cells. No significant difference in killing was observed (Supporting Information Figure 7E). Similarly, CD19-SynNotch PDbody-CAR T cells cocultured with a CD19-positive but PD-L1-negative K562 cell line (expressing CD19-P2A-firefly luciferase) did not elicit nonspecific cytotoxicity (Supporting Information Figure 7F). Having established these controls, we performed the killing assays with varying E/T ratios and verified that CD19-SynNotch PDbody-CAR T cell was effective in eradicating the MDA-MB-231 target cells expressing tCD19 (Figure 4C). We then mixed CD19^+^ and CD19^–^ MDA-MB-231 cells at a ratio of 1:1 to examine whether a subset of cancer cells can be introduced with the clinically validated CD19 to serve as “training centers” and trigger the production of PDbody-CARs in T cells for the eradication of the whole population of MDA-MB-231 cells which universally express a high level of PD-L1.^44,48^ It is expected that the half CD19^+^ MDA-MB-231 cells will train and activate CD19-SynNotch to induce PDbody CAR production in T cells to target PD-L1 on both CD19^+^ and CD19^–^ MDA-MB-231 cell populations (Figure 4D). Indeed, both CD19^+^ and CD19^–^ MDA-MB-231 cells were attacked in all the coculture groups with CD19-SynNotch PDbody-CAR T cells demonstrating the highest level of killing (Figures 4E). Moreover, PDbody-CAR can completely clear both CD19^+^ and CD19^–^ MDA-MB-231 cells in 24 h with an E/T ratio of 3:1, whereas Mb-FG-EVO CAR and Mb-G9 CAR were unable to eradicate the cancer cells even after 48 h of coculture, although Mb-FG-EVO CAR showed higher cytotoxicity than that of Mb-G9 CAR (Supporting Information Figure 8A). This result is exciting, as PDbody-CAR integrated with CD19-SynNotch can produce CAR T cells to target solid cancer cells engineered to express clinically validated antigens (*e.g*., CD19), albeit partially and heterogeneously. These CD19-expressing cancer cells can serve as “training centers” to induce PDbody-CAR, which can attack the whole population of cancer cells at the tumor site expressing PD-L1, which is not tumor-specific.

To investigate the minimal “on-target off-tumor” effects associated with our CD19 SynNotch PDbody-CAR design, we cocultured CD19^+^ MDA-MB-231 cells with our CD19-SynNotch PDbody-CAR Jurkat cells for a period of 24 h. After this incubation, Jurkat cells were separated from the adherent MDA-MB-231 cells, and the expression of the PDbody CAR was monitored over the next 24 h (Supporting Information Figure 8B). The result showed that the expression of CAR reduced to basal levels within 6 h following the detachment of the Jurkat cells from the MDA-MB-231 cells. This rapid reversion to baseline levels of CAR expression is a critical observation, indicating a minimized risk of off-tumor toxicity, particularly against bystander cells located at a distance from the primary tumor site. Such findings underscore the safety and specificity of our engineered CAR T cells in targeting tumor cells while mitigating collateral damage to healthy tissues.

### SynNotch-Gated PDbody-CAR In Vivo

**Figure 5:**
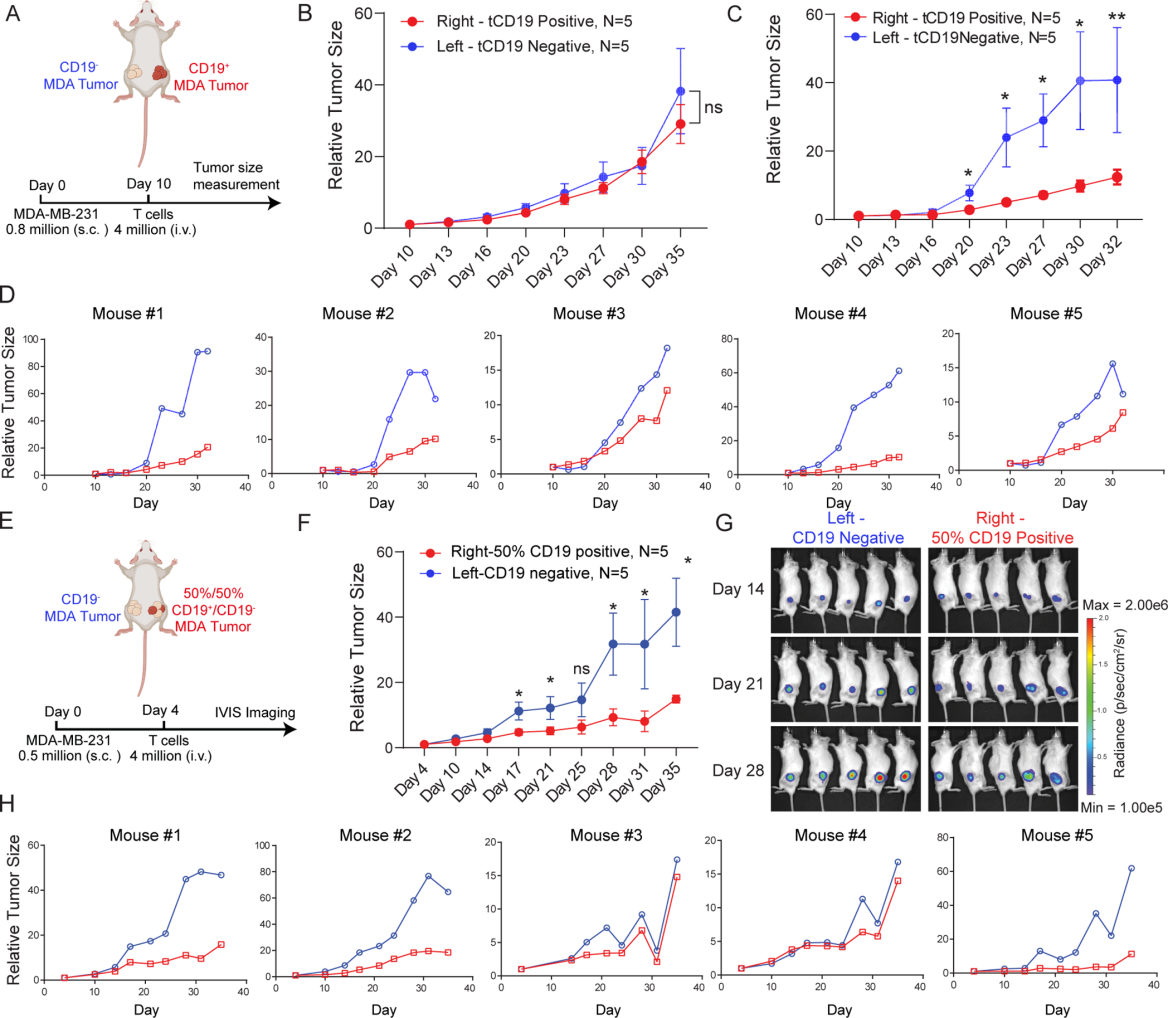
**CD19-SynNotch PDbody-CAR suppresses tumor growth *in vivo*.** (A) Experimental design for *in vivo* bilateral tumor mouse model. MDA-MB-231 cancer cells were injected into mice and were allowed to grow for 10 days at which point CD19-SynNotch PDbody-CAR T cells were injected intravenously. Tumor growth was monitored via caliper measurement. (B) Relative tumor sizes over time in the nontreatment group. CD19^–^ and CD19^+^ tumor growth rates are represented as lines in blue and red, respectively. (C, D) Relative averaged tumor sizes (C) and tumor size of individual mice (D) over time in the treatment group. CD19^–^ and CD19^+^ tumor growth rates are represented as lines in blue and red, respectively. (E) Experimental design for *in vivo* bilateral tumor mouse model with 1:1 CD19^+^/CD19^–^ cancer mixtures. MDA-MB-231 cancer cells (Left: CD19^–^ only; Right: 1:1 CD19^+^/CD19^–^ mixture) were injected into mice and were allowed to grow for 4 days, at which point CD19-SynNotch PDbody-CAR T cells were injected intravenously. Tumor growth was monitored via IVIS measurement. (F) Relative averaged tumor sizes over time for (E). The growth curves of CD19^–^ cancer cells on the left and right sides are represented as lines in blue and red, respectively. (G) IVIS images of the tumor burden over time. (H) Relative tumor size measurements for individual mice in *in vivo* 50% PD-L1 positive/negative bilateral tumor model.

After verifying the function of the CD19-SynNotch PDbody-CAR system *in vitro*, it was tested in a bilateral tumor NOD scid gamma mouse (NSG) mouse model. Equal numbers of CD19^+^ and CD19^–^ MDA-MB-231 cells with high PD-L1 expression were injected into the right and left flanks, respectively, of 5 mice, and tumor size was monitored via caliper measurement every 3–4 days (Figure 5A). We first compared the tumor growth without T cell injection, and the result showed a similar growth rate between the CD19^+^ and CD19^–^ tumors (Figure 5B). In the experimental group, after 10 days of tumor growth, CD19-SynNotch PDbody-CAR T cells were injected intravenously. Results indicated that tumor growth of CD19^+^ tumors was significantly slowed compared to that of CD19^–^ tumors (Figure 5C,D), indicating that the CD19-SynNotch PDbody-CAR T cells can robustly suppress the tumor growth of CD19^+^ tumors *in vivo*.

To demonstrate that CD19-SynNotch PDbody-CAR T cells can kill tumors partially engineered with clinically validated CD19, another *in vivo* experiment was performed with only 50% of the target cancer cells expressing CD19 to serve as training centers to induce PDbody CAR expression in T cells. Equal numbers of CD19^–^ MDA-MB-231 and a 1:1 CD19^+^/CD19^–^ MDA-MB-231 mixture were injected into the left and right flanks, respectively. CD19-SynNotch PDbody-CAR T cells were intravenously injected 4 days after tumor implantation (Figure 5E). CD19^–^ tumor growth was monitored via renilla luciferase luminescence and normalized to the first day of the luminescence measurement. Results indicate that from day 17 onward, the growth of the CD19^–^ cancer cells was significantly suppressed on the right side where there were 50% CD19^+^ cancer cells serving as “training centers” to induce PDbody CAR production to target both CD19^–^ and CD19^+^ cancer cells at the tumor site (Figure 5F–H). Overall, these results suggest that the integration of SynNotch and PDbody CAR can be applied to add an additional level of control over cytotoxicity, allowing PDbody CAR T cells to target *in vivo* solid tumors engineered to express clinically validated antigens.

## Conclusions

In this study, we integrated computational modeling-based rational design together with directed evolution utilizing yeast display and high-throughput screening of mutation libraries to develop PDbody. This integration is key to developing PDbody, a monobody product with a high affinity for PD-L1 and a preference for the acidic tumor microenvironment. Based on a single-domain monobody derived from human fibronectin,^26−28^ PDbody should have high stability with less immunogenicity. PDbody was further utilized to generate PDbody-CAR, which recognizes PD-L1 on the cancer cell surface for killing. Integrated with SynNotch recognizing an introduced and clinically validated antigen CD19 expressed on a subset of cancer cells as “training centers”, CD19-SynNotch PDbody-CAR T cells were further applied to target the whole population of cancer cells expressing PD-L1. This IF THEN gate integrating CD19-SynNotch and PDbody-CAR should enhance the specificity of T cell killing and potentially minimize on-target off-tumor toxicity in adoptive cell therapy, as PDbody-CAR is induced and maintained mainly in the proximity of “training centers” where SynNotch engages the CD19 antigen.

PDbody was engineered through a combination of rational design and directed evolution to bind to PD-L1 with a micromolar affinity. Interestingly, pH 7.4 PBS buffer was insufficient to detect PD-L1 binding for yeast staining during the initial experiments. For this reason, pH 6.5 MES buffer was used for directed evolution. A possible drawback of lower pH screening is that it can be a poor representation of physiological conditions. Taking this into consideration, the pH was kept above 6.5, which is around the p*K*_a_ of histidine. Below pH 6.0, histidine becomes biprotonated and positively charged, which may change the overall charge state and conformation of the monobody, potentially causing misrepresentation of the physiological conformation. Nonetheless, a benefit of lower pH screening is that it can leverage the acidic nature of the tumor microenvironment to increase the targeting specificity and minimize toxic targeting of healthy tissues at physiological pH.

PDbody was engineered from Mb-G9 with an affinity of 4.75 μM under physiological pH solutions, which is better than that of wild-type PD-1.^50,51^ We hypothesized that this moderate affinity could protect against off-tumor toxicity, particularly against normal tissues or organs expressing low levels of PD-L1. From the killing assays performed in this study, complete killing was observed at 3:1 and 5:1 E/T ratios but was incomplete at 1:1 E/T ratio (Figure 4C). This suggests that CD19-SynNotch PDbody-CAR T cells could be more locally cytotoxic at the tumor site and less cytotoxic if they migrated away to other locations where they would be scattered with decaying CAR expression,^44^ which should be beneficial to mitigate the off-tumor toxicity of standard CAR T cells. Nevertheless, CD19-SynNotch PDbody-CAR T cells were able to suppress cancer cells *in vitro* and *in vivo*. This protein, in addition to its neutralization of immunosuppressive checkpoint to promote CAR killing efficacy, possesses several advantages afforded by the single domain monobody scaffold, such as ease of folding due to the lack of disulfide bonds, small molecular weight, and human origin, thus adding another tool to the immunotherapeutic arsenal.

To add another layer of precise control to the PDbody-CAR and prevent on-target off-tumor toxicity, its expression was controlled by a CD19-SynNotch receptor. Without CD19 or PD-L1 expression on target cancer cells, cytotoxicity was not observed (Supporting Information Figure 7E,F). Upon SynNotch engagement, CAR is produced at the proximity of tumor regions, where the clinically validated antigen can be potentially introduced to express in a subset of cancer cells as “training centers” through genetic modification. Notably, CD19 is expressed on the surface of B cells; therefore, leaky CAR expression due to B cell exposure is a concern. Fortunately, B cell aplasia is clinically manageable,^64,65^ and in clinical trials, fludarabine and cyclophosphamide have been established to pretreat patients for lymphodepletion before CAR T application^66^ to avoid the issue of nonspecific CD19-SynNotch activation by CD19^+^ B cells.

We acknowledge that there have been previously published, engineered single-domain CARs targeting PD-L1, namely, a constitutively expressed PD-L1-targeting nanobody CAR;^24^ however, we believe the CD19-SynNotch PDbody-CAR improves over the previously published nanobody CAR for several reasons. First, as a human-based protein scaffold, the monobody has less potential risk for immunogenicity.^30,31^ Second, the addition of the CD19-SynNotch IF THEN gate not only mitigates the effect of on-target antitumor toxicity but also improves the proliferative capability of the T cells. PD-L1 can be expressed on T cells themselves; thus, premature activation or fratricide is a potential issue for constitutively expressed CARs. Indeed, we noticed that constitutively expressed PDbody-CAR primary T cells could kill themselves. Therefore, the addition of SynNotch not only mitigates off-tumor toxicity but also can increase the overall efficacy of CAR treatment by suppressing autotoxicity. We noticed that the CD19-SynNotch PDbody-CAR only needed 3:1 E/T ratio to eliminate the MDA-MB-231 target *in vitro*, but the nanobody CAR needed a 10:1 E/T ratio to eliminate the B16 target. Thus, addition of SynNotch to any CAR system can be an effective means to reduce pretumor exhaustion.

While our current study is focused on the directed evolution of nanoscale, decent PD-L1 binders and their application in CD19-SynNotch PDbody-CAR T cells, we are enthusiastic about the prospect of future research that delves into the integration of nanodelivery technologies. Specifically, we can foresee the future integration of nanoparticles for the tumor-specific delivery of antigens,^67,68^ or *in vivo* genetic manipulation of CAR-T cells^69−71^ would further achieve better T cell control, improve T cell function, and enhance the tumor elimination efficacy of our CD19-SynNotch PDbody-CAR T cells.

## Methods

### Molecular Cloning

Plasmids were generated using the Gibson Assembly (NEB, E2611L), T4 ligation (NEB, M0202L), and golden gate assembly (Thermo Fisher Scientific, FERER0452). PCR was performed using Q5 DNA polymerase (NEB, M0491) and synthesized primers (Integrated DNA Technologies). Constructs were verified by Sanger sequencing (Azenta) (Supporting Information Tables 1 & 2).

### Protein Purification of Recombinant PD-L1

An expression vector pEF-Bos containing PD-L1 was transfected into HEK 293T Lenti-X 293T cells with Lipofectamine 3000 (Life Technologies, L3000). Cells were cultured in Advanced DMEM (ThermoFisher, 124291015) with 1X Penicillin/Streptomycin (Fisher Scientific, 15140122) and 2X Glutamax (Fisher Scientific, 35050061). Media were collected after 2 days of culture. Protease cocktail inhibitors were added (Millipore Sigma, 11697498001), and proteins were extracted and concentrated using 3 kDa Amicon centrifugal units (Millipore Sigma, UFC800396) through 5 successive 25 min spindowns at 4 °C and 7830 rpm. PD-L1 was then purified via its coupled 6xHis tag with a Ni-NTA beads (Qiagen, 30210) nickel column and biotinylated using BirA biotin-protein ligase standard reaction kit (Avidity, BirA500). Buffer exchange to PBS was performed using 10 kDa snakeskin dialysis tubing for 24 h with 2 L of PBS (500 mL for 3 h, 500 mL for 5 h, and 1 L for 16 h). Total protein concentration was determined using Bio-Rad Protein Assay Dye Reagent Concentrate (Bio-Rad, #5000006). Anti PD-L1 antibody was used in a Western blot to verify protein identity (eBioscience, 14-5983-82). Streptavidin Alexa Fluor 488 (Invitrogen, S11223) immunoblot staining was used to verify biotinylation.

### Protein Purification of Biotinylated Monobodies

Monobody constructs with biotin targeting sites were cloned into the pRSET vector and then transformed into BL21 (DE3) cells. Cells were cultured in LB Amp and then induced in 0.5 mM IPTG overnight at 16 °C. Cells were lysed in B-PER (ThermoFisher, 78243) with one Complete, EDTA-free Protease Inhibitor Cocktail tablet (Millipore Sigma, 04693132001) and 100 μM PMSF. The supernatant was filtered, and then monobody proteins were purified using nickel column purification (Qiagen, 30210). Biotinylation was performed using a BirA biotin-protein ligase standard reaction kit (Avidity, BirA500). Buffer exchange to PBS was performed using 3 kDa snakeskin dialysis tubing for 24 h with 2 L of PBS (500 mL for 3 h, 500 mL for 5 h, and 1 L for 16 h). Total protein concentration was determined using the Bradford Assay. Streptavidin Alexa Fluor 488 immunoblot staining was used to verify the protein identity and biotinylation.

### Yeast Culture

*Saccharomyces cerevisiae* EBY100 (a GAL1-AGA1::URA3 ura3–52 trp1 leu2Δ1 his3Δ200 pep4::HIS2 prbΔ1. 6R can1 GAL) was used for the yeast surface display. EBY100 was cultured in rich media (YPD) until transformation with yeast display plasmid pYD1 (ThermoFisher, V835-01). For selection, yeast cells were grown in synthetic complete medium minus tryptophan (SC-Trp with 2% (w/v) glucose). To induce monobody expression, yeast cells were induced in galactose media (SC-Trp with 2% (w/v) galactose.

### K_D_ Measurement Via Flow Cytometry

The protein–protein dissociation constant *K*_D_ of monobody was measured using yeast surface display as described.^53^ Antigen concentrations ranging from 10 pM to 5 μM were applied to label 1 × 10^7^ induced yeast cells. Biotinylated PD-L1 was incubated with yeast cells at room temperature for 45 min to reach binding equilibrium, and then the resulting cells were stained with streptavidin-PE for 15 min at 4 °C. Nonlinear least-squares regression was used to calculate the *K*_D_ to be 47 nM. Flow cytometry data was analyzed using Flowjo Software (Flowjo, LLC).

### K_D_ Measurement Via Biolayer Interferometry

Binding kinetics of biotinylated monobody and PD-L1 were measured using Bio-Layer Interferometry. 9 μM biotinylated monobody was loaded onto the streptavidin biosensor for 2 min, and PD-L1 association was then measured for 2 min, followed by dissociation observed for another 2 min. Data was exported into Matlab, and nonlinear regression was used to determine *k*_on_, *k*_off_, and *K*_D_ values according to the procedures as reported earlier.^72^

### Simulations of Molecular Dynamics for Monobody Optimization

Starting from Mb-G9 (PDB: 1ttg), we grafted the CDR3 loop of known PD-L1 binder (PDB: 5jds) into the FG loop of the Mb while preserving the CDR3-PDL1 interface. The resulting Mb-035 was solvated in a water box with 1 nm padding with 150 mM NaCl. Counterions were added to neutralize the net charge of the system. All molecular dynamics simulations were performed with OpenMM using a Langevin integrator with a friction coefficient of 1.0/ps, the Amber ff14SB force field, and the TIP3P water model.^73^ The system was minimized twice with 1000 max iterations and 5 kJ/mol tolerance. In the first run, 1 kJ/Å^2^ harmonic restraints were applied to non-hydrogen atoms in G9NbFG and all backbone atoms in PDL1. In the second minimization, 1 kJ/Å^2^ harmonic restraints were applied to all of the backbone atoms. After minimization, the system was gradually heated to 300 K from 25 K in increments of 25 K using an integration time step of 2 fs/step and 50,000 steps with protein restrained with 1 kJ/Å^2^ harmonic restraints. Following heating, the system was equilibrated for 50,000 steps with backbone restrained before a final equilibration of 500,000 steps with no restraints. After system preparation and equilibration, we performed a 2 μs production simulation with the Geodesic BAOAB integrator from OpenMM tools.^74^ The resulting trajectory was superposed onto the first frame, and conformations were clustered into 3 conformational states using spectral clustering of atomic coordinates. Hydrogen bonding and atomic contacts (radius of 3.5 Å) were calculated for each frame.

### Library Construction

Site-saturated libraries were constructed in the BC loop (residues 26–30) and KN035 inserted into the FG loop regions of the monobody. To prepare plasmids for the Golden Gate Assembly, ESP3I sites were cloned into the pYD1 vector. To create regions of genetic variance, DNA library synthesis via primer annealing was performed using NNK primers, where N represents an equimolar mixture of A, T, G, and C nucleotides, and K represents an equimolar ratio of T and G nucleotides. Golden Gate Assembly was performed, transformed into MegaX DH10B Electrocomp cells (ThermoFisher C640003), and then purified with Qiagen HiSpeed Plasmid Maxi kit. Purified DNA was transformed into EBY100 cells according to the following protocol as previously reported.^75^

### FACS Screening of Monobody Library

BC and FG loop libraries were sorted using BD FACSAria. To induce monobody expression, yeast cells were cultured in 2% galactose-containing synthetic complete medium minus tryptophan. Cells were induced at 20 °C and shaken at 250 rpm for 48 h. After induction, 5 × 10^7^ cells were stained with 1 μM biotinylated PD-L1, αV5 Alexa Fluor 647 (ThermoFisher, 451098) at 1:100 v/v, and propidium iodide (ThermoFisher, P1304MP) at 1:750 v/v for 90 min. After primary staining, cells were stained with PE-SA (BD Biosciences, 554061) for 30 min at a concentration of 1:100 (v/v) for the FG loop library and 1:1000 (v/v) for the BC loop library. Washing and staining were performed in PBS buffer with 0.1% bovine serum albumin at pH 7.4 for the FG loop library and PBS buffer with 0.1% bovine serum albumin at pH 6.5 titrated with 10 M HCl. Buffers were filtered with 0.22 μm filters for sterility.

### General Mammalian Cell Culture

Human embryonic kidney (HEK293T) and MDA-MB-231 cells were cultured in Dulbecco’s Modified Eagle Medium (DMEM) (Gibco, 11995115) with 10% fetal bovine serum (FBS) (Gibco, 10438026) and 1% penicillin-streptomycin (P/S) (Gibco, 15140122). Jurkat and K562 cells were cultured in Roswell Park Memorial Institute Medium (RPMI 1640) (Gibco, 22400105) with 10% FBS and 1% P/S. Primary human T cells were cultured in complete RPMI 1640 supplemented with 100 U mL^–1^ of recombinant human IL-2 (PeproTech, 200-02). All cell types were cultured at 37 °C in a humidified 5% CO_2_ incubator.

### Isolation and Transduction of Primary Human T Cells

Human peripheral blood mononuclear cells were isolated from buffy coats from the San Diego Blood Bank with a lymphocyte separation medium (Corning, 25-072-CV). Primary human T cells were isolated using a Pan T Cell Isolation Kit (Milteenyi, 130-096-535). Following isolation, T cells were stimulated with Dynabeads Human T-Expander CD3/CD28 (ThermoFisher, 11141D) at a ratio of 1:1 Dynabeads per T cell. 48 h after Dynabead stimulation, cells were transduced on Retronectin-coated (Takara, T100B) plates with concentrated lentivirus at a multiplicity of infection 5 per construct. Six days after infection Dynabeads were magnetically removed, T cells were stained with anti-myc Alexa Fluor 488 (Cell Signaling Technology, 9B11), and FACS sorted with a SONY SH800.

### Cytotoxicity Assay

MDA-MB-231 target cell lines were generated through lentiviral transduction and subsequent sorting with a SONY SH800 sorter. For cytotoxicity assays, 2.5 × 10^4^ CD19 positive and 2.5 × 10^4^ CD19 negative MDA-MB-231 cells were cocultured with 2.5 × 10^5^ CD19-SynNotch monobody-CAR T cells in 150 μL of RPMI for 24 h. Bioluminescence measurements were taken using the Dual Glo Luciferase Assay kit (Promega, model E2920). Cytotoxicity was measured by calculating the percent difference in luminescence of SynNotch T cells versus that of target cancer cells only.

### In Vivo Bilateral Tumor Model

Animal experiments were performed in accordance with Protocol S15285, which was approved by the UCSD Institutional Animal Care and Use Committee (IACUC). All researchers involved in this study complied with animal-use guidelines and ethical regulations. 6-week-old NOD scid gamma (NSG) mice, purchased from UCSD Animal Care Program (ACP), were used in the study. Five mice were subcutaneously injected with 8 × 10^5^ CD19^+^ MDA-MB-231 cells in the right flank and 8 × 10^5^ CD19^–^ MDA-MB-231 cells in the left flank. Ten days after tumor injection, 4 × 10^6^ CD19-SynNotch PDbody-CAR T cells were intravenously injected. Tumor volume was then measured twice a week via caliper measurement. Volume was calculated using the equation (*l* × *w* × *w*)/2, where *l* is the longest length of the tumor and *w* is the length of the tumor perpendicular to *l*.

For the 50% CD19^+^/CD19^–^ experiment, 6-week-old NOD scid gamma (NSG) mice, purchased from UCSD Animal Care Program (ACP), were used in the study. Five mice were subcutaneously injected with 2.5 × 10^5^ CD19^+^ MDA-MB-231 and 2.5 × 10^5^ CD19^–^ MDA-MB-231 cells in the right flank and 5 × 10^5^ CD19^–^ MDA-MB-231 cells in the left flank. Four days after tumor injection, 4 × 10^6^ CD19-SynNotch PDbody-CAR T cells were intravenously injected. The growth of CD19^–^ cancer cells on both sides was imaged using IVIS 10 min after Coelentarizine injection (GoldBio, CZ2.5) following the manufacturer’s protocol.

### Statistical Analysis

Statistical analysis was performed using Prism software and described in the figure legends. For *in vivo* studies, tumor volume was measured with the exponential growth law, and its growth rate was computed at the time *t* as log(*V*(*t*)/*V*(0)), where *V*(*t*) was tumor volume at time *t* and *V*(0) was the tumor volume at time 0 before T cell treatment. For luminescence measurements, the growth rate was calculated as log(Relative Luminescence). Regression analysis was performed on tumor growth rates with a randomized block design for each day separately, followed by residual analysis for checking model assumptions. Specifically, for each day, a linear regression model was built, *y* = mouse + treatment + error, where response *y* was the tumor growth rate for a mouse receiving one of the two treatments and the error term represented the experimental error. Here, each mouse formed a block of size two. The randomized block design was effective in eliminating mouse-to-mouse variation. Statistical tests were conducted using ANOVA and F tests. *P* values based on two-sided *t* tests were computed to determine the significance of the treatment effect. Residual analysis of the model confirmed the accuracy of the model assumptions. Statistical analysis was performed using R (http://www.r-project.org/), a free software environment for statistical computing and graphics.

## Supporting Information Available

Supporting Information Figures 1–8 and Supporting Information Tables 1–2 are provided to include the plasmid constructs and the gene fragments information (PDF)
