# Supplementary material for: Engineering a Programmed Death-Ligand 1-Targeting Monobody Via Directed Evolution for SynNotch-Gated Cell Therapy
Source: ACS Nano. 2024 Mar 8;18(11):8531–45. doi: 10.1021/acsnano.4c01597 (PMC10958600; doi:10.1021/acsnano.4c01597)
Supplement: Supplementary file 1 — nn4c01597_si_001.pdf [file nn4c01597_si_001.pdf]

Supporting Information

Engineering a Programmed Death-Ligand 1-Targeting Monobody *via* Directed Evolution for SynNotch-Gated Cell Therapy

Linshan Zhu[1,2,#], Chi-Wei Man[3,#], Reed E.S. Harrison[1], Zhuohang Wu[2], Praopim Limsakul[1,5,6], Qin Peng[1,4], Matthew Hashimoto[1], Anthony P. Mamaril[1], Hongquan Xu[7], Longwei Liu[1,2*], and Yingxiao Wang[1,2*]

Affiliations:

1. Department of Bioengineering & Institute of Engineering in Medicine, University of California, San Diego, La Jolla, CA 92093, USA
2. Alfred E. Mann Department of Biomedical Engineering, University of Southern California, Los Angeles, CA 90089, USA
3. Department of Chemistry and Biochemistry, University of California, San Diego, La Jolla, CA 92093, USA
4. Institute of Systems and Physical Biology, Shenzhen Bay Laboratory, Shenzhen, 518132, P.R. China
5. Division of Physical Science, Faculty of Science, Prince of Songkla University, Hat Yai, Songkhla 90110, Thailand
6. Center of Excellence for Trace Analysis and Biosensor, Prince of Songkla University, Hat Yai, Songkhla 90110, Thailand
7. Department of Statistics, University of California, Los Angeles, CA, 90095, USA

[#]These authors contributed to the work equally

*To whom correspondence should be addressed:

Yingxiao Wang, Ph. D.

E-mail: ywang283@usc.edu

Longwei Liu, Ph. D.

E-mail: longweil@usc.edu

This file includes:

Supplementary Figures 1-8

Supplementary Tables 1-2

Supplementary Table 1: Construct list

Supplementary Table 2: Codon-optimized gene fragments

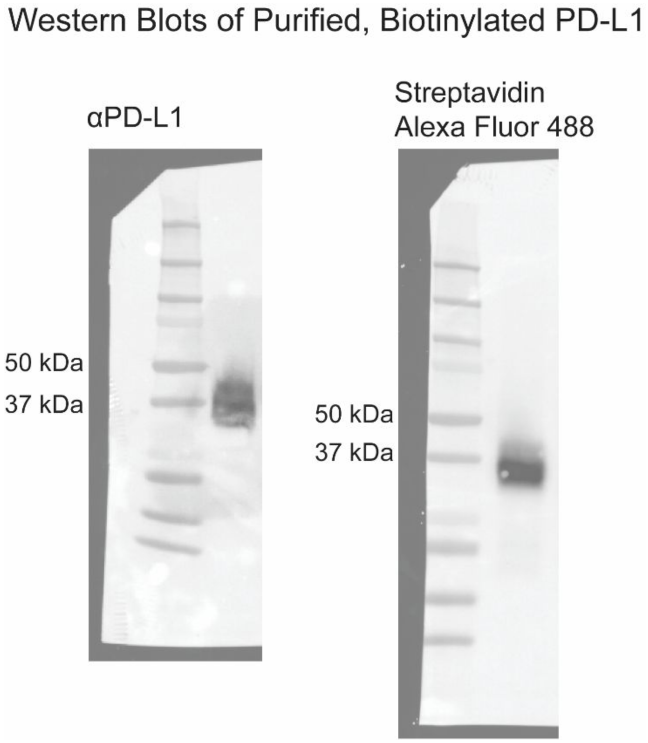

Supplementary Figure 1. Western blot of purified and biotinylated PD-L1. The left image shows a Western blot of PD-L1 using αPD-L1 antibody which was visualized with goat α-mouse Horseradish Peroxidase (HRP). The right shows the detection of biotinylated PD-L1 using streptavidin Alexa Fluor 488 conjugate.

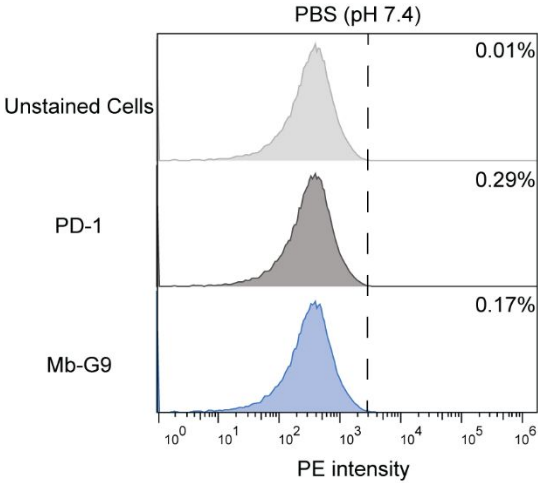

Supplementary Figure 2. Mb-G9 has weak binding to PD-L1 under physiological condition. PD-L1 binding in Phosphate-Buffered Saline (PBS) (0.5% w/v Bovine Serum Albumin (BSA), pH 7.4). PD-L1 binding of Mb-G9 is shown in blue, PD-L1 binding of wildtype PD-1 is shown in dark gray, and unstained cells are shown in light gray.

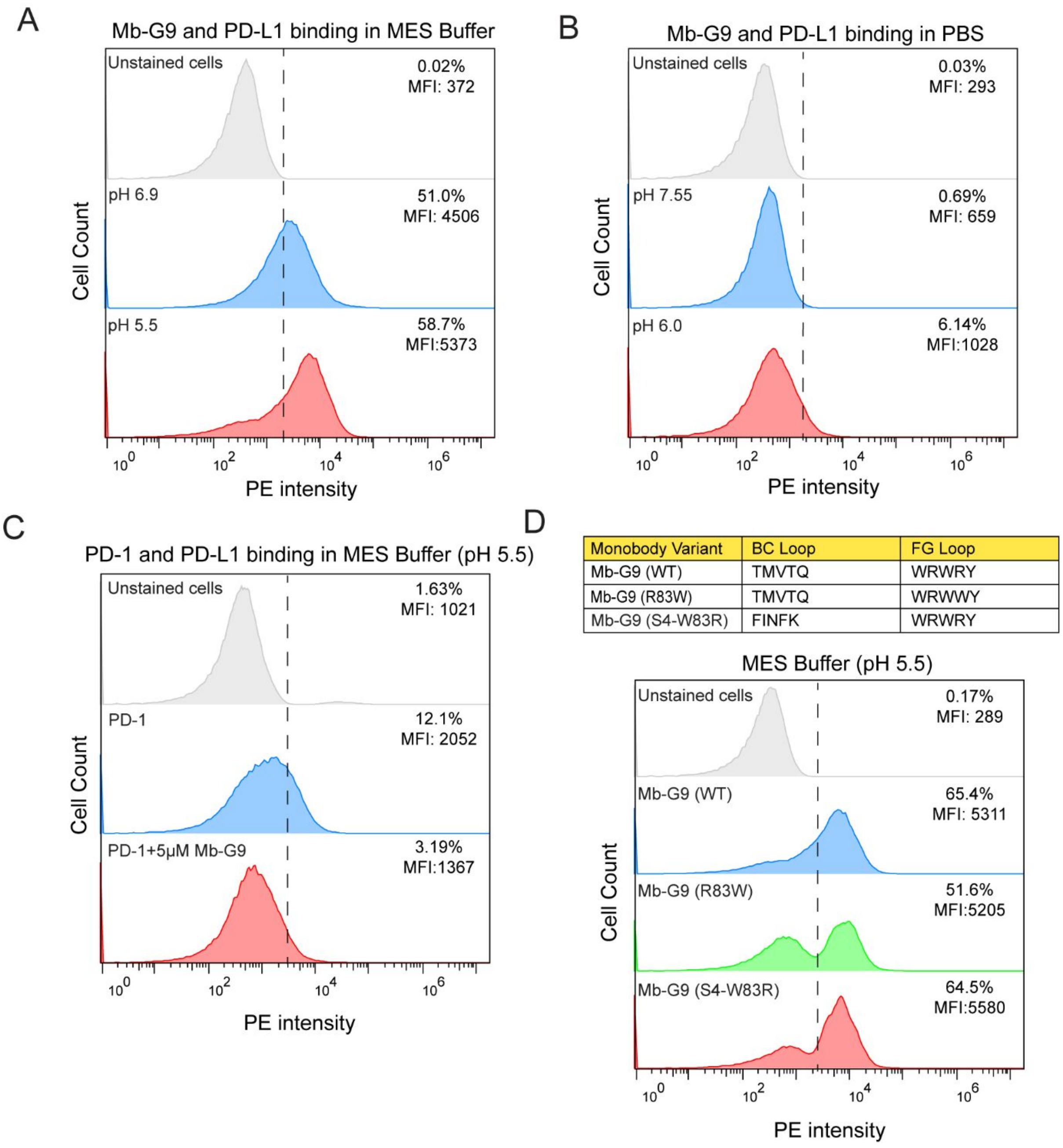

Supplementary Figure 3. PD-L1 binding of Mb-G9 in different buffer conditions and pH's. Induced yeast cells expressing Mb-G9 were stained with 5 µM biotinylated PD-L1 and streptavidin-phycoerythrin. **(A)** Mb-G9 and PD-L1 binding in 2-(N-morpholino)ethanesulfonic acid (MES) buffer (20 mM MES Sodium, 150 mM NaCl, 0.0005%Tween) at pH 5.5 (red) and pH 6.9 (blue). **(B)** Mb-G9 and PD-L1 binding in PBS buffer (0.5% w/v BSA) at pH 6.0 (red) and pH 7.55 (blue). Unstained cells are shown in light gray.

(C) Mb-G9 was added to see whether it actively inhibited WT PD-1 binding. Shown in blue is PD-L1 binding (5 μM) of WT PD-1 and shown in red is PD-L1 binding (5 μM) of WT PD-1 with 5 μM Mb-G9 added and shown in gray is unstained cells. **(D)** PD-L1 binding of monobody variants with different loop modifications. The modifications are shown in the table, and the staining results are shown in the figure: Monobody-G9 (WT) in blue, Monobody-G9 (R83W) in green, and Monobody-S4 (W83R) in red. PD-L1 binding was not significantly affected. Gray is unstained cells.

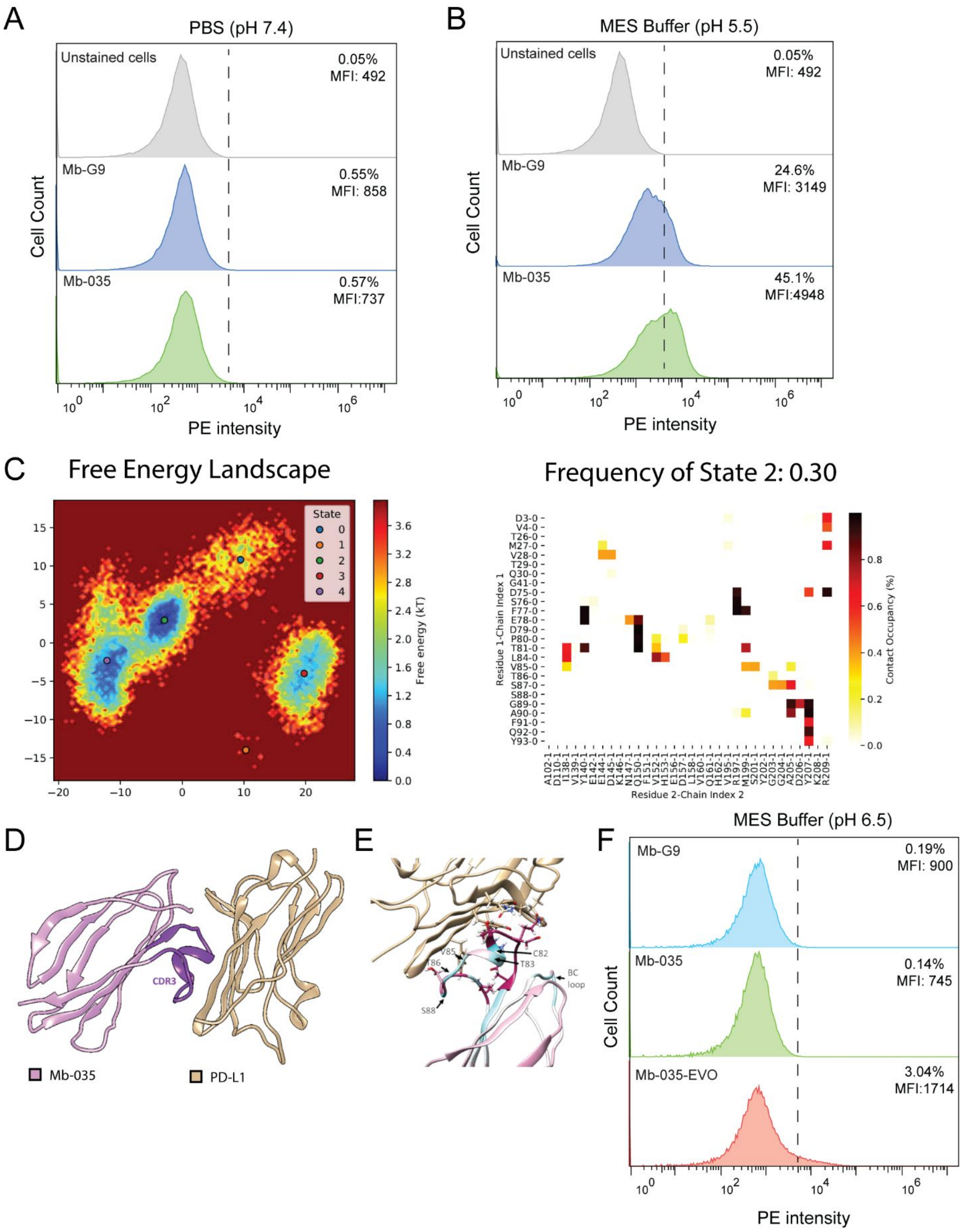
A
PBS (pH 7.4)
Unstained cells
0.05%
MFI: 492
Mb-G9
0.55%
MFI: 858
Mb-035
0.57%
MFI:737
Cell Count
PE intensity
B
MES Buffer (pH 5.5)
Unstained cells
0.05%
MFI: 492
Mb-G9
24.6%
MFI: 3149
Mb-035
45.1%
MFI:4948
Cell Count
PE intensity
C
Free Energy Landscape
State
Free energy (kT)
Frequency of State 2: 0.30
Residue 1-Chain Index 1
Residue 2-Chain Index 2
Contact Occupancy (%)
D3-0 V4-0 T26-0 M27-0 V28-0 T29-0 Q30-0 G41-0 D75-0 S76-0 F77-0 E78-0 D79-0 P80-0 T81-0 L84-0 V85-0 T86-0 S87-0 S88-0 G89-0 A90-0 F91-0 Q92-0 Y93-0
A102-1 D110-1 I138-1 V139-1 Y140-1 E142-1 E144-1 D145-1 K146-1 N147-1 Q150-1 F151-1 V152-1 H153-1 E156-1 D157-1 L158-1 V160-1 Q161-1 H162-1 V195-1 R197-1 M199-1 S201-1 Y202-1 G203-1 G204-1 A205-1 D206-1 Y207-1 K208-1 R209-1
D
CDR3
Mb-035
PD-L1
E
V85
T86
S88
C82
T83
BC loop
F
MES Buffer (pH 6.5)
Mb-G9
0.19%
MFI: 900
Mb-035
0.14%
MFI: 745
Mb-035-EVO
3.04%
MFI:1714
Cell Count
PE intensity

Supplementary Figure 4. Characterization and directed evolution of Mb-035. (A) and **(B)** Induced yeast cells expressing Mb-G9 or Mb-035 were stained with 5 μM biotinylated PD-L1 in PBS (A) or MES buffer (B) and streptavidin-phycoerythrin (SA-PE). PD-L1 binding of Mb-G9 is shown in blue, PD-L1 binding of Mb-035 is shown in green, and PD-L1 binding of unstained cells is shown in light gray. **(C)** Left: Energy landscape from a 2 μs simulation of Mb-035 bound to PD-L1 with 5 conformational states labeled. Because state 2 was one of the most energetically favorable, it was further analyzed. Right: Contact occupancies for interactions between Mb-035 and PD-L1. An occupancy of 1 indicates that the contact is observed in every instance of the conformational state. **(D)** Model of Mb-035 (magenta) binding to PD-L1 (light brown). Grafted Mb-035 loop is shown in dark purple. **(E)** Molecular dynamics simulation of Mb-035 (magenta and cyan) binding to PD-L1 (light brown). Optimizable residues are labeled and shown in cyan. **(F)** Induced yeast cells expressing Mb-G9, Mb-035, and Mb-035-EVO were stained with 5 μM biotinylated PD-L1 and streptavidin-PE in pH 6.5 MES buffer. PD-L1 binding of Mb-G9 is shown in blue, PD-L1 binding of Mb-035 is shown in green, and PD-L1 binding of Mb-035-EVO cells is shown in light gray.

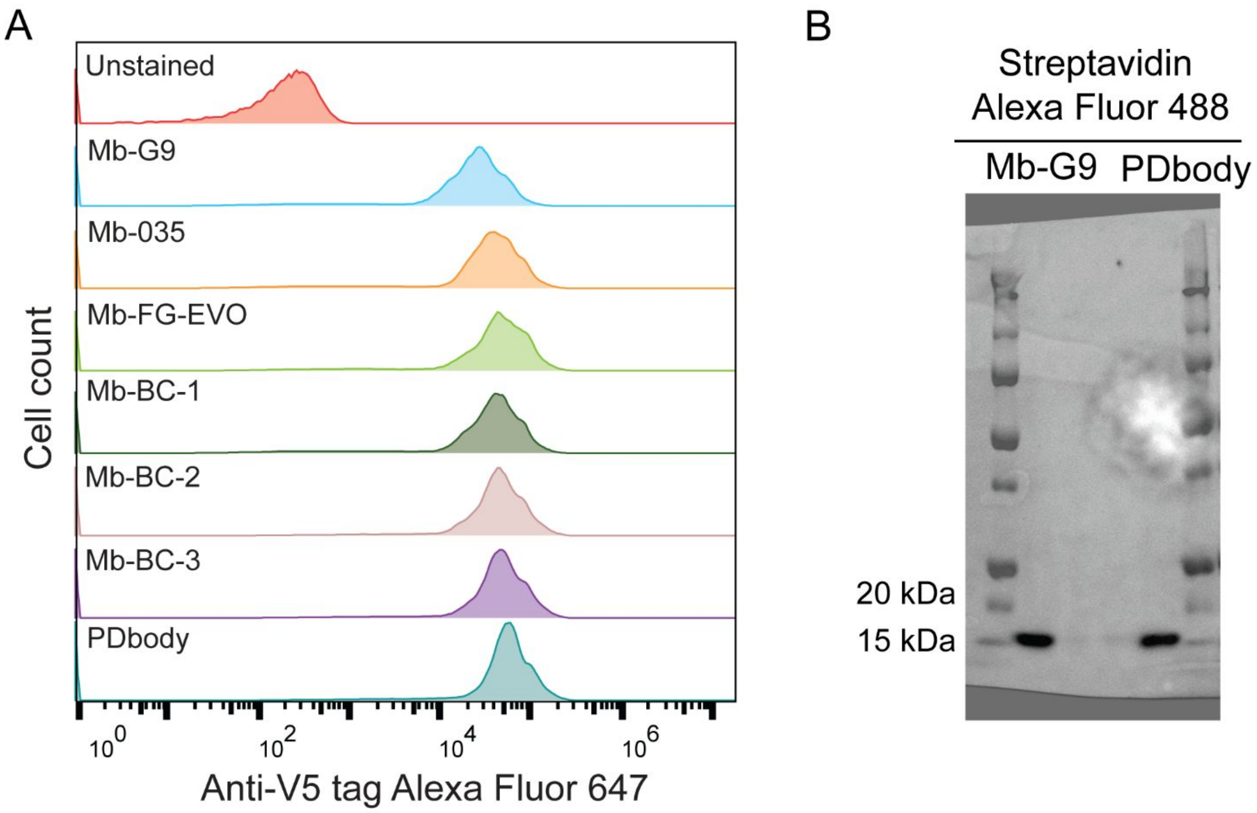

Supplementary Figure 5. Monobody variants expression levels comparison. (A) Expression levels of induced, yeast-displayed monobody variants. (B) Western blot of purified and biotinylated monobodies. The left sample lane shows Mb-G9, and the right lane shows PDbody. Proteins were detected using streptavidin Alexa Fluor 488 conjugate.

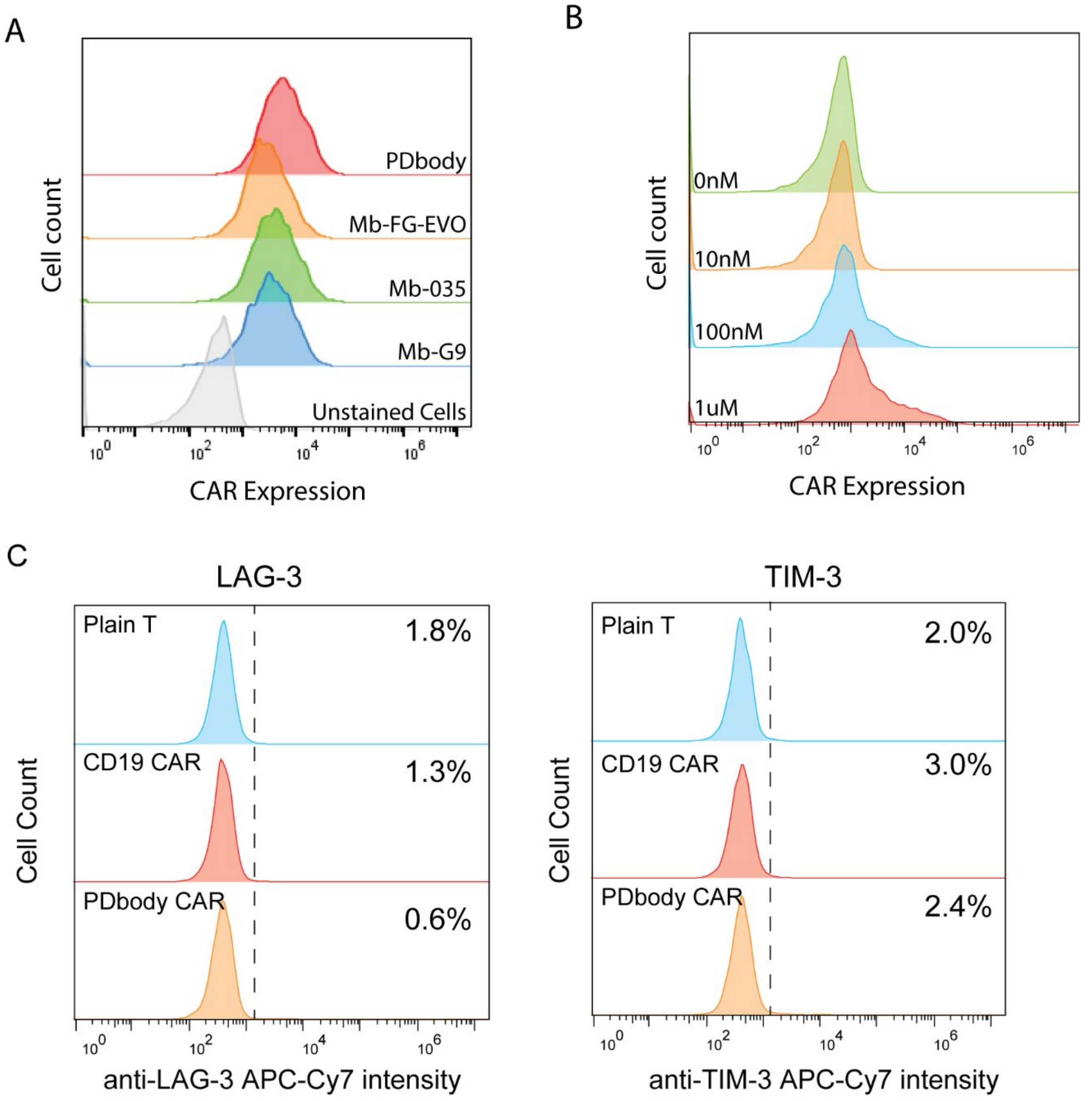

Supplementary Figure 6. Characterization of monobody CAR expression in Jurkat cells and T cell exhaustion markers. (A) FACS-sorted Jurkat cells expressing monobody CARs were labeled with anti-myc Alexa Fluor 647. CAR expression level of Mb-G9-CAR is shown in blue; CAR expression level of Mb-035-CAR is shown in green; CAR expression level of Mb-FG-EVO CAR is shown in orange, and CAR expression level of PDbody-CAR is shown in red. **(B)** PD-L1 binding of PDbody CARs. 10 nM, 100 nM, and 1 μM biotinylated PD-L1 and streptavidin-PE were used to stain Jurkat-displayed PDbody CARs in pH 7.4 PBS buffer. **(C)** Lymphocyte-activation gene 3 (LAG-3) and T-cell Immunoglobulin and

Mucin-domain containing-3 (TIM-3) staining of plain, CD19 CAR and PDbody CAR T cells 7 days after lentivirus transduction.

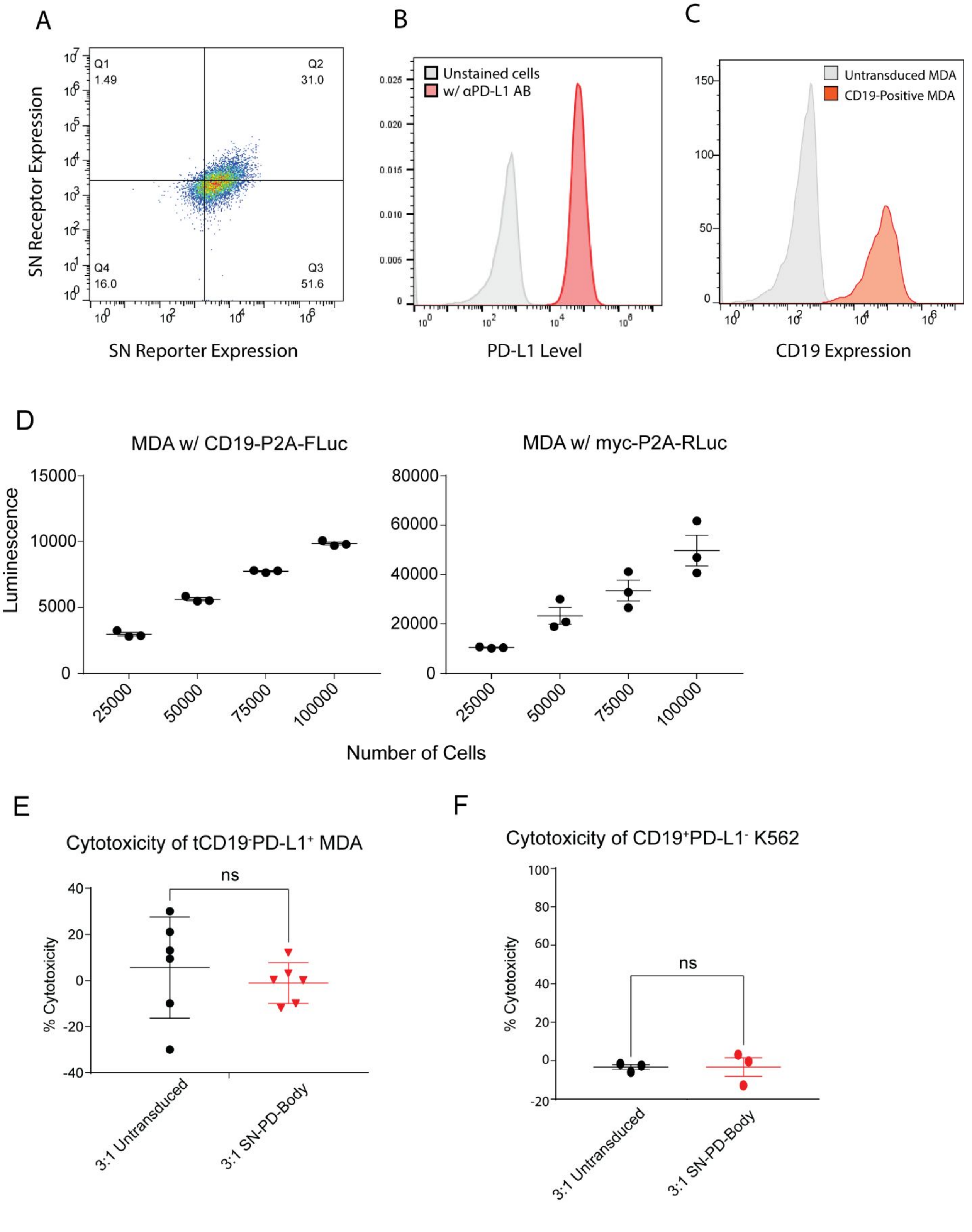
A
B
C
Q1
1.49
Q2
31.0
Q4
16.0
Q3
51.6
SN Receptor Expression
SN Reporter Expression
Unstained cells
w/ αPD-L1 AB
PD-L1 Level
Untransduced MDA
CD19-Positive MDA
CD19 Expression
D
MDA w/ CD19-P2A-FLuc
MDA w/ myc-P2A-RLuc
Luminescence
Number of Cells
E
Cytotoxicity of tCD19-PD-L1+ MDA
% Cytotoxicity
ns
3:1 Untransduced
3:1 SN-PD-Body
F
Cytotoxicity of CD19+PD-L1- K562
% Cytotoxicity
ns
3:1 Untransduced
3:1 SN-PD-Body

Supplementary Figure 7. Verification of the SynNotch CAR system. (A) Expression levels of SynNotch constructs in T cells. αCD19 SynNotch monobody CAR constructs were detected *via* anti-myc Alexa Fluor 647 staining for the SynNotch receptor and mCherry expression for the SynNotch reporter. **(B)** Measurement of PD-L1 expression in MDA-MB-231 cells. MDA-MB-231 cells were stained with anti-PD-L1 Allophycocyanin (APC) antibody to verify PD-L1 expression levels. **(C)** Measurement of CD19 expression level in MDA cell line. Lentivirally transduced and FACS-sorted MDA cells were stained with anti-CD19 Alexa Fluor 647 antibody to determine CD19 levels. **(D)** Bioluminescence standard measurement of MDA-MB-231 target cell lines. Luminescence of standard numbers of MDA-MB-231 cells were measured using (left) firefly luciferase substrate for the $CD19^+$ cell line (MDA-MB-231 w/ CD19-P2A- Firefly Luciferase) and (right) renilla luciferase substrate for the $CD19^-$ cell line (MDA-MB-231 w/ myc-P2A-Renilla luciferase). **(E)** Killing assay was performed against $CD19^-$ $PD\text{-}L1^+$ MDA-MB-231 cells at a 3:1 E:T ratio. Cytotoxicity of plain T cells is shown in black, and cytotoxicity of CD19-SynNotch PDbody-CAR T cells is shown in red. **(F)** Killing assay was performed against $CD19^+$ $PD\text{-}L1^-$ K562 cells at a 1:1 E:T ratio. Cytotoxicity of plain T cells is shown in black, and cytotoxicity of CD19-SynNotch PDbody-CAR T cells is shown in red.

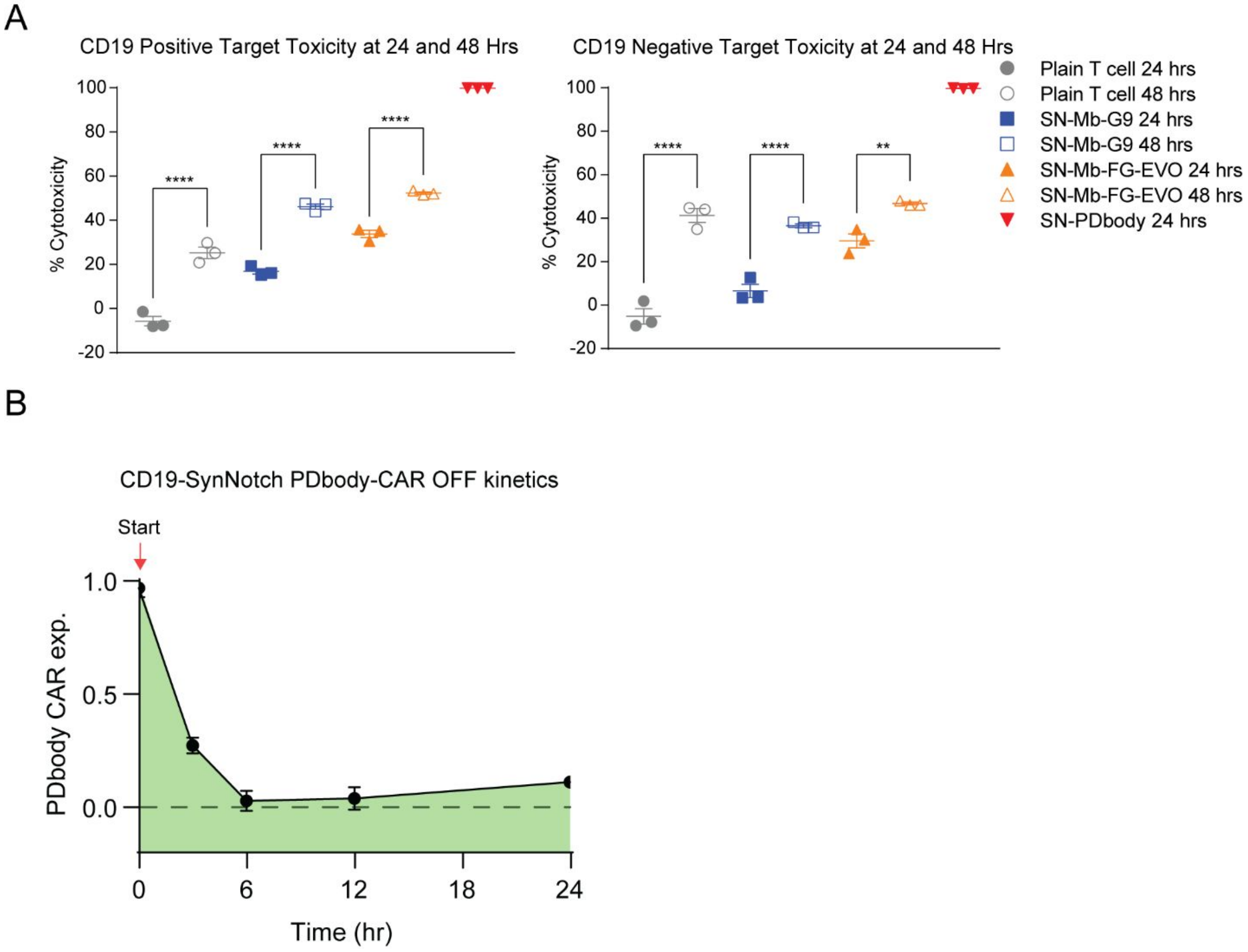

Supplementary Figure 8. (A) Killing assay of CD19-SynNotch monobody-CARs at 24 and 48 hrs. Luminescence was measured 24 and 48 hrs after co-culture for each of the different groups. Measurements were performed in triplicate. Cytotoxicity of plain T cells is shown in light gray, cytotoxicity of CD19-SynNotch Mb-G9-CAR T cells is shown in blue, cytotoxicity of CD19-SynNotch Mb-FG-EVO-CAR T cells is shown in orange, and cytotoxicity of CD19-SynNotch PDbody-CAR T cells is show in red. **(B)** Time course of the surface PDbody-CAR expression in CD19-SynNotch PDbody-CAR Jurkat cells after removal from $CD19^{+}$MDA-MB-231 cells. Jurkat T cells expressing the IF THEN circuit were stimulated for 24 hr by $CD19^{+}$MDA-MB-231 cells. START indicates time at which cells were removed from the target $CD19^{+}$MDA-MB-231 cells and the expression level of PDbody-CAR were monitored.

Supplementary Table 1: Construct list

Plasmid ID	Detailed Cloning Description	Source
pEFBos	Mammalian expression vector (P_{hEF1a}-stuffer-poly(A)).	Mizushima *et al.*, 1990
pEFBosPDL1	Mammalian expression vector of human PD-L1 (P_{hEF1a}-PDL1-BTS-6xHisTag). This insert was formed by PCR of GBlock PDL1 with P1 and P2. The vector was formed by digestion of pEFBos with XbaI and BamHI. T4 ligation was used to form the contruct.	This paper
pRSETbMbG9	Bacterial expression vector (P_{T7}-6xHis-MbG9) coding for Mb-G9.	Limsakul et al., 2018
pRSETbMbG9BTS	Bacterial expression vector (P_{T7}-6xHis-MbG9-BTS) coding for Mb-G9-BTS for protein purification. This construct was cloned by Gibson assembly with one insert. BTS insertion was generated by annealing P3 and P4. Vector backbone was amplified with oligos P5-P6 on pRSETbMbG9.	This paper
pYD1MbG9R83W	Yeast display vector (PT7-Aga2-Xpress-MbG9R83W-V5-6xHis) for displaying MbG9 R83W.	Limsakul *et al.*, 2018
pYD1MbG9S4W83R	Yeast display vector (P_{T7}-Aga2-Xpress-MbG9S4W83R-V5-6xHis) for displaying Mb-G9 (S4-W83R).	Limsakul *et al.*, 2018
pYD1MbG9	Yeast display vector (P_{T7}-Aga2-Xpress-MbG9-V5-6xHis) for displaying Mb-G9.	Huang *et al.*, 2011
pYD1	Yeast display vector (PT7-Aga2-Xpress-MCS-V5-6xHis) with multiple cloning site.	Kieke *et al.*, 1997 Addgene (no. 73447)
pYD1hPD1	Yeast display vector (P_{T7}-Aga2-Xpress-PD1-V5-6xHis) for displaying PD-1. This construct was cloned by golden gate assembly. PD-1 IgV insert was amplified with P7-P8 oligos on GBlockPD1. pYD1 was digested with ESP3I.	This paper
pYD1Mb035	Yeast display vector (P_{T7}-Aga2-Xpress-Mb035-V5-6xHis) for displaying Mb-035. This construct was cloned by Gibson assembly with no insert. Vector	This paper

	backbone was amplified with P9-P10 oligos on pYD1MbG9.	
pYD1MbFGEVO	Yeast display vector (P_{T7}-Aga2-Xpress-MbFGEVO-V5-6xHis) for displaying Mb-FG-EVO. This was obtained by yeast miniprep of a library clone.	This paper
pYD1MbBC1	Yeast display vector (P_{T7}-Aga2-Xpress-MbBC1-V5-6xHis) for displaying Mb-BC-1. This was obtained by yeast miniprep of a library clone.	This paper
pYD1MbBC2	Yeast display vector (P_{T7}-Aga2-Xpress-MbBC2-V5-6xHis) for displaying Mb-BC-2. This was obtained by yeast miniprep of a library clone.	This paper
pYD1MbBC3	Yeast display vector (P_{T7}-Aga2-Xpress-MbBC3-V5-6xHis) for displaying Mb-BC-3. This was obtained by yeast miniprep of a library clone.	This paper
pYD1PDBody	Yeast display vector (P_{T7}-Aga2-Xpress-PDBody-V5-6xHis) for displaying PDbody. This was obtained by yeast miniprep of a library clone.	This paper
pRSETbPDBodyBTS	Bacterial expression vector (P_{T7}-6xHis-PDBody-BTS) coding for PDbody-BTS. This construct was cloned by Gibson assembly with one insert. Vector backbone was amplified with P11-P12 oligos on pRSETbMbG9BTS. Insert was amplified by annealing P13 and P14.	This paper
pHRPGKLoxHmChyLoxPcMycCARv2	Mammalian expression of CD19 CAR (P_{PGK}-LoxH-mCherry-LoxP-myc-CD19CAR).	Wu *et al.*, 2021
pHR_PGK_antiCD19_synNotch_Gal4VP64	Mammalian SynNotch receptor construct targeting CD19 antigen (P_{PGK}-CD19scFv-SynNotch-Gal4DBD-VP64).	Morsut *et al.*, 2016 Addgene (no. 79125)
pHRMbG9CAR	Mammalian expression of constitutive Mb-G9 CAR (P_{PGK}-MbG9-CD28-41BB-CD3z). This construct was cloned by Gibson assembly with two inserts. Vector backbone was amplified with P15-P16 oligos on pHR_PGK_antiCD19_synNotch_Gal4VP64. Insert 1 was amplified by P17-P18 oligos on pHRPGKLoxHmChyLoxPcMycCARv2. Insert 2 was amplified with P19-P20 oligos on pYD1MbG9.	This paper
pHRMb035CAR	Mammalian expression of constitutive Mb-035 CAR (P_{PGK}-Mb035-CD28-41BB-CD3z). This construct was cloned by Gibson assembly with two inserts. Vector backbone was amplified with P15-P16 oligos on pHR_PGK_antiCD19_synNotch_Gal4VP64. Insert 1 was amplified by P17-P18 oligos on	This paper

	pHRPGKLoxHmChyLoxPcMycCARv2. Insert 2 was amplified with P19-P20 oligos on pYD1Mb035.	
pHRMbFGEVOCAR	Mammalian expression of constitutive Mb-FG-EVO CAR (P_{PGK}-MbFGEVO-CD28-41BB-CD3z). This construct was cloned by Gibson assembly with two inserts. Vector backbone was amplified with P15-P16 oligos on pHR_PGK_antiCD19_synNotch_Gal4VP64. Insert 1 was amplified by P17-P18 oligos on pHRPGKLoxHmChyLoxPcMycCARv2. Insert 2 was amplified with P19-P20 oligos on pYD1MbFGEVO.	This paper
pHRPDBodyCAR	Mammalian expression of constitutive PDbody CAR (P_{PGK}-PDBody-CD28-41BB-CD3z). This construct was cloned by Gibson assembly with two inserts. Vector backbone was amplified with P15-P16 oligos on pHR_PGK_antiCD19_synNotch_Gal4VP64. Insert 1 was amplified by P17-P18 oligos on pHRPGKLoxHmChyLoxPcMycCARv2. Insert 2 was amplified with P19-P20 oligos on pYD1PDBody.	This paper
pHRPGKCD19	Mammalian expression of constitutive CD19 (P_{PGK}-CD19). This construct was cloned using by Gibson assembly with one insert. Vector backbone was amplified with P21-P22 oligos on pHR_PGK_antiCD19_synNotch_Gal4VP64. Insert was amplified with P23-24 on GBlock-CD19.	This paper
pHRCD19P2AFLuc	Mammalian target cell display vector (P_{PGK}-CD19-P2A-FLuc). This construct was cloned by Gibson assembly with one insert. Vector backbone was amplified with P25-P26 oligos on pHRPGKCD19. Insert was amplified with P27-P28 on GBlockP2AFLuc.	This paper
pHRmycP2ARLuc	Mammalian target cell display vector (P_{PGK}-myc-P2A-RLuc). This construct was cloned by Gibson assembly with one insert. Vector backbone was amplified with P29-P30 oligos on pHR_PGK_antiCD19_synNotch_Gal4VP64. Insert was amplified with P27-P31 on GBlockP2ARLuc.	This paper
pHR_Gal4UAS_tBFP_PGK_mCherry	Mammalian SynNotch reporter construct with inducible expression of tag BFP (4xGal4UAS-P_{CMV}-tBFP-P_{PGK}-mCherry).	Morsut *et al.*, 2016 Addgene (no. 79130)
pNCS-Antares	Bacterial expression of BRET reporter (P_{T7}-NanoLuciferase-CyOFP).	Chu *et al.*, 2016 Addgen

		e (no. 74279)
pHRNC3SNRepmCherry	Mammalian SynNotch reporter construct with inducible expression of NC3 BRET reporter with constitutive mCherry (4xGal4UAS-P_{CMV}-NanoLuc-P_{PGK}-mCherry). This construct was cloned by Gibson assembly with one insert. Vector backbone was amplified with P32-P33 oligos on pHR_Gal4UAS_tBFP_PGK_mCherry. Insert was amplified with P34-P35 on pNCS-Antares.	This paper
pHRNC3SNRepeGFP	Mammalian SynNotch reporter construct with inducible expression of NC3 BRET reporter with constitutive eGFP (4xGal4UAS-P_{CMV}-NanoLuc-CyOFP-P_{PGK}-mCherry). This construct was cloned by Gibson assembly with one insert. Vector backbone was amplified with P36-P37 oligos on pHRNC3SNRepmCherry. Insert was amplified with P38-P39 on GBlockeGFP.	This paper
pHRMbG9CARSNRep	Mammalian SynNotch reporter construct with inducible expression of Mb-G9 CAR with constitutive mCherry (4xGal4UAS-P_{CMV}-MbG9CAR-P_{PGK}-mCherry). This construct was cloned by Gibson assembly with one insert. Vector backbone was amplified with P40-P41 oligos on pHR_Gal4UAS_tBFP_PGK_mCherry. Insert was amplified with P42-P43 on pHRMbG9CAR.	This paper
pHRMbFGEVOCARSNRep	Mammalian SynNotch reporter construct with inducible expression of Mb-FG-EVO CAR with constitutive mCherry (4xGal4UAS-P_{CMV}-MbFGEVO-P_{PGK}-mCherry). This construct was cloned by Gibson assembly with one insert. Vector backbone was amplified with P40-P41 oligos on pHR_Gal4UAS_tBFP_PGK_mCherry. Insert was amplified with P42-P43 on pHRMbFGEVOCAR.	This paper
pHRPDBodyCARSNREP	Mammalian SynNotch reporter construct with inducible expression of PDbody CAR with constitutive mCherry (4xGal4UAS-P_{CMV}-PDBody-P_{PGK}-mCherry). This construct was cloned by Gibson assembly with one insert. Vector backbone was amplified with P40-P41 oligos on pHR_Gal4UAS_tBFP_PGK_mCherry. Insert was amplified with P42-P43 on pHRPDBodyCAR.	This paper
pYD1FGLoopLibrary	Yeast display vector (P_{T7}-Aga2-Xpress-MonobodyFGLoopLibrary-V5-6xHis) for library screening. First, Golden Gate template was generated by Gibson assembly with one insert. Vector backbone was amplified with P44-P45 oligos on pYD1Mb035. Insert was amplified with P46-P47 oligos on pYD1Mb035. Golden Gate insert was generated by annealing of P48-	This paper

	P49 oligos. Gibson product and insert were digested with ESP3I and annealed.	
pYD1BCLoopLibrary	Yeast display vector (P_{T7}-Aga2-Xpress-MonobodyBCLoopLibrary-V5-6xHis) for library screening. First, Golden Gate template was generated by Gibson assembly with one insert. Vector backbone was amplified with P50-P51 oligos on pYD1MbFGEVO. Insert was amplified with P52-53 oligos on pYD1MbFGEVO. Golden Gate insert was generated by annealing of P54-55 oligos. Gibson product and insert were digested with ESP3I and annealed.	This paper

Supplementary Table 2: Codon-optimized gene fragments

**Gene Name**	**Gene Fragment Sequence (5'-3')**
PDL1	TCTATCTAGAGCCACCATGAGGATATTTGCTGTCTTTATATTCATGACCTACTGG<br>CATTTGCTGAACGCATTTACTGTCACGGTTCCCAAGGACCTATATGTGGTAGAG<br>TATGGTAGCAATATGACAATTGAATGCAAATTCCCAGTAGAAAAACAATTAGA<br>CCTGGCTGCACTAATTGTCTATTGGGAAATGGAGGATAAGAACATTATTCAATT<br>TGTGCATGGAGAGGAAGACCTGAAGGTTCAGCATAGTAGCTACAGACAGAGGG<br>CCCGGCTGTTGAAGGACCAGCTCTCCCTGGGAAATGCTGCACTTCAGATCACAG<br>ATGTGAAATTGCAGGATGCAGGGGTGTACCGCTGCATGATCAGCTATGGTGGTG<br>CCGACTACAAGCGAATTACTGTGAAAGTCAATGCCCCATACAACAAAATCAAC<br>CAAAGAATTTTGGTTGTGGATCCAGTCACCTCTGAACATGAACTGACATGTCAG<br>GCTGAGGGCTACCCCAAGGCCGAAGTCATCTGGACAAGCAGTGACCATCAAGT<br>CCTGAGTGGTAAGACCACCACCACCAATTCCAAGAGAGAGGAGAAGCTTTTCA<br>ATGTGACCAGCACACTGAGAATCAACACAACAACTAATGAGATTTTCTACTGCA<br>CTTTTAGGAGATTAGATCCTGAGGAAAACCATACAGCTGAATTGGTCATCCCAG<br>AACTACCTCTGGCACATCCTTCCGGATCACTGCATCATATTCTGGATGCACAGA<br>AAATGGTGTGGAATCATCGTTAAGGATCCAAGC

PD1	ATGCAGATCCCACAGGCGCCCTGGCCAGTCGTCTGGGCGGTGCTACAACTGGGC<br>TGGCGGCCAGGATGGTTCTTAGACTCCCCAGACAGGCCCTGGAACCCCCCCACC<br>TTCTCCCCAGCCCTGCTCGTGGTGACCGAAGGGGACAACGCCACCTTCACCTGC<br>AGCTTCTCCAACACATCGGAGAGCTTCGTGCTAAACTGGTACCGCATGAGCCCC<br>AGCAACCAGACGGACAAGCTGGCCGCCTTCCCCGAGGACCGCAGCCAGCCCGG<br>CCAGGACTGCCGCTTCCGTGTCACACAACTGCCCAACGGGCGTGACTTCCACAT<br>GAGCGTGGTCAGGGCCCGGCGCAATGACAGCGGCACCTACCTCTGTGGGGCCA<br>TCTCCCTGGCCCCCAAGGCGCAGATCAAAGAGAGCCTGCGGGCAGAGCTCAGG<br>GTGACAGAGAGAAGGGCAGAAGTGCCCACAGCCCACCCCAGCCCCTCACCCAG<br>GCCAGCCGGCCAGTTCCAAACCCTGGTGGTTGGTGTCGTGGGCGGCCTGCTGGG<br>CAGCCTGGTGCTGCTAGTCTGGGTCCTGGCCGTCATCTGCTCCCGGGCCGCACG<br>AGGGACAATAGGAGCCAGGCGCACCGGCCAGCCCCTGAAGGAGGACCCCTCAG<br>CCGTGCCTGTGTTCTCTGTGGACTATGGGGAGCTGGATTTCCAGTGGCGAGAGA<br>AGACCCCGGAGCCCCCCGTGCCCTGTGTCCCTGAGCAGACGGAGTATGCCACCA<br>TTGTCTTTCCTAGCGGAATGGGCACCTCATCCCCCGCCCGCAGGGGCTCAGCTG<br>ACGGCCCTCGGAGTGCCCAGCCACTGAGGCCTGAGGATGGACACTGCTCTTGGC<br>CCCTC

CD19	ATGCCACCTCCTCGCCTCCTCTTCTTCCTCCTCTTCCTCACCCCCATGGAAGTCA<br>GGCCCGAGGAACCTCTAGTGGTGAAGGTGGAAGAGGGAGATAACGCTGTGCTG<br>CAGTGCCTCAAGGGGACCTCAGATGGCCCCACTCAGCAGCTGACCTGGTCTCGG<br>GAGTCCCCGCTTAAACCCTTCTTAAAACTCAGCCTGGGGCTGCCAGGCCTGGGA<br>ATCCACATGAGGCCCCTGGCATCCTGGCTTTTCATCTTCAACGTCTCTCAACAGA<br>TGGGGGGCTTCTACCTGTGCCAGCCGGGGCCCCCCTCTGAGAAGGCCTGGCAGC<br>CTGGCTGGACAGTCAATGTGGAGGGCAGCGGGGAGCTGTTCCGGTGGAATGTTT<br>CGGACCTAGGTGGCCTGGGCTGTGGCCTGAAGAACAGGTCCTCAGAGGGCCCC<br>AGCTCCCCTTCCGGGAAGCTCATGAGCCCCAAGCTGTATGTGTGGGCCAAAGAC<br>CGCCCTGAGATCTGGGAGGGAGAGCCTCCGTGTGTCCCACCGAGGGACAGCCT<br>GAACCAGAGCCTCAGCCAGGACCTCACCATGGCCCCTGGCTCCACACTCTGGCT<br>GTCCTGTGGGGTACCCCCTGACTCTGTGTCCAGGGGCCCCCTCTCCTGGACCCAT<br>GTGCACCCCAAGGGGCCTAAGTCATTGCTGAGCCTAGAGCTGAAGGACGATCG<br>CCCGGCCAGAGATATGTGGGTAATGGAGACGGGTCTGTTGTTGCCCCGGGCCAC<br>AGCTCAAGACGCTGGAAAGTATTATTGTCACCGTGGCAACCTGACCATGTCATT<br>CCACCTGGAGATCACTGCTCGGCCAGTACTATGGCACTGGCTGCTGAGGACTGG<br>TGGCTGGAAGGTCTCAGCTGTGACTTTGGCTTATCTGATCTTCTGCCTGTGTTCC<br>CTTGTGGGCATTCTTCATCTTCAAAGAGCCCTGGTCCTGAGGAGGAAAAGAAAG<br>CGAATGACTGACCCCACCAGGAGATTCTTCAAAGTGACGCCTCCCCCAGGAAGC<br>GGGCCCCAGAACCAGTACGGGAACGTGCTGTCTCTCCCCACACCCACCTCAGGC<br>CTCGGACGCGCCCAGCGTTGGGCCGCAGGCCTGGGGGGCACTGCCCCGTCTTAT<br>GGAAACCCGAGCAGCGACGTCCAGGCGGATGGAGCCTTGGGGTCCCGGAGCCC<br>GCCGGGAGTGGGCCCAGAAGAAGAGGAAGGGGAGGGCTATGAGGAACCTGAC<br>AGTGAGGAGGACTCCGAGTTCTATGAGAACGACTCCAACCTTGGGCAGGACCA<br>GCTCTCCCAGGATGGCAGCGGCTACGAGAACCCTGAGGATGAGCCCCTGGGTC<br>CTGAGGATGAAGACTCCTTCTCCAACGCTGAGTCTTATGAGAACGAGGATGAAG<br>AGCTGACCCAGCCGGTCGCCAGGACAATGGACTTCCTGAGCCCTCATGGGTCAG<br>CCTGGGACCCCAGCCGGGAAGCAACCTCCCTGGGGTCCCAGTCCTATGAGGATA<br>TGAGAGGAATCCTGTATGCAGCCCCCCAGCTCCACTCCATTCGGGGCCAGCCTG<br>GACCCAATCATGAGGAAGATGCAGACTCTTATGAGAACATGGATAATCCCGAT<br>GGGCCAGACCCAGCCTGGGGAGGAGGGGGCCGCATGGGCACCTGGAGCACCAG<br>GTGA

	GGAAGCGGAGCTACTAACTTCAGCCTGCTGAAGCAGGCTGGAGACGTGGAGGA
	GAACCCTGGACCTATGGAAGATGCCAAAAACATTAAGAAGGGCCCAGCGCCAT
	TCTACCCACTCGAAGACGGGACCGCCGGCGAGCAGCTGCACAAAGCCATGAAG
	CGCTACGCCCTGGTGCCCGGCACCATCGCCTTTACCGACGCACATATCGAGGTG
	GACATTACCTACGCCGAGTACTTCGAGATGAGCGTTCGGCTGGCAGAAGCTATG
	AAGCGCTATGGGCTGAATACAAACCATCGGATCGTGGTGTGCAGCGAGAATAG
	CTTGCAGTTCTTCATGCCCGTGTTGGGTGCCCTGTTCATCGGTGTGGCTGTGGCC
	CCAGCTAACGACATCTACAACGAGCGCGAGCTGCTGAACAGCATGGGCATCAG
	CCAGCCCACCGTCGTATTCGTGAGCAAGAAAGGGCTGCAAAAGATCCTCAACG
	TGCAAAAGAAGCTACCGATCATACAAAAGATCATCATCATGGATAGCAAGACC
	GACTACCAGGGCTTCCAAAGCATGTACACCTTCGTGACTTCCCATTTGCCACCC
	GGCTTCAACGAGTACGACTTCGTGCCCGAGAGCTTCGACCGGGACAAAACCATC
	GCCCTGATCATGAACAGTAGTGGCAGTACCGGATTGCCCAAGGGCGTAGCCCTA
	CCGCACCGCACCGCTTGTGTCCGATTCAGTCATGCCCGCGACCCCATCTTCGGC
	AACCAGATCATCCCCGACACCGCTATCCTCAGCGTGGTGCCATTTCACCACGGC
	TTCGGCATGTTCACCACGCTGGGCTACTTGATCTGCGGCTTTCGGGTCGTGCTCA
	TGTACCGCTTCGAGGAGGAGCTATTCTTGCGCAGCTTGCAAGACTATAAGATTC
	AATCTGCCCTGCTGGTGCCCACACTATTTAGCTTCTTCGCTAAGAGCACTCTCAT
	CGACAAGTACGACCTAAGCAACTTGCACGAGATCGCCAGCGGCGGGGCGCCGC
	TCAGCAAGGAGGTAGGTGAGGCCGTGGCCAAACGCTTCCACCTACCAGGCATC
	CGCCAGGGCTACGGCCTGACAGAAACAACCAGCGCCATTCTGATCACCCCCGA
	AGGGGACGACAAGCCTGGCGCAGTAGGCAAGGTGGTGCCCTTCTTCGAGGCTA
	AGGTGGTGGACTTGGACACCGGTAAGACACTGGGTGTGAACCAGCGCGGCGAG
	CTGTGCGTCCGTGGCCCCATGATCATGAGCGGCTACGTTAACAACCCCGAGGCT
	ACAAACGCTCTCATCGACAAGGACGGCTGGCTGCACAGCGGCGACATCGCCTA
	CTGGGACGAGGACGAGCACTTCTTCATCGTGGACCGGCTGAAGAGCCTGATCA
	AATACAAGGGCTACCAGGTAGCCCCAGCCGAACTGGAGAGCATCCTGCTGCAA
	CACCCCAACATCTTCGACGCCGGGGTCGCCGGCCTGCCCGACGACGATGCCGGC
	GAGCTGCCCGCCGCAGTCGTCGTGCTGGAACACGGTAAAACCATGACCGAGAA
	GGAGATCGTGGACTATGTGGCCAGCCAGGTTACAACCGCCAAGAAGCTGCGCG
	GTGGTGTTGTGTTCGTGGACGAGGTGCCTAAAGGACTGACCGGCAAGTTGGACG
P2AFL	CCCGCAAGATCCGCGAGATTCTCATTAAGGCCAAGAAGGGCGGCAAGATCGCC
uc	GTGTAA

P2ARLuc	GGAAGCGGAGCTACTAACTTCAGCCTGCTGAAGCAGGCTGGAGACGTGGAGGA GAACCCTGGACCTATGGCTTCCAAGGTGTACGACCCCGAGCAACGCAAACGCA TGATCACTGGGCCTCAGTGGTGGGCTCGCTGCAAGCAAATGAACGTGCTGGACT CCTTCATCAACTACTATGATTCCGAGAAGCACGCCGAGAACGCCGTGATTTTTC TGCATGGTAACGCTGCCTCCAGCTACCTGTGGAGGCACGTCGTGCCTCACATCG AGCCCGTGGCTAGATGCATCATCCCTGATCTGATCGGAATGGGTAAGTCCGGCA AGAGCGGGAATGGCTCATATCGCCTCCTGGATCACTACAAGTACCTCACCGCTT GGTTCGAGCTGCTGAACCTTCCAAAGAAAATCATCTTTGTGGGCCACGACTGGG GGGCTTGTCTGGCCTTTCACTACTCCTACGAGCACCAAGACAAGATCAAGGCCA TCGTCCATGCTGAGAGTGTCGTGGACGTGATCGAGTCCTGGGACGAGTGGCCTG ACATCGAGGAGGATATCGCCCTGATCAAGAGCGAAGAGGGCGAGAAAATGGTG CTTGAGAATAACTTCTTCGTCGAGACCATGCTCCCAAGCAAGATCATGCGGAAA CTGGAGCCTGAGGAGTTCGCTGCCTACCTGGAGCCATTCAAGGAGAAGGGCGA GGTTAGACGGCCTACCCTCTCCTGGCCTCGCGAGATCCCTCTCGTTAAGGGAGG CAAGCCCGACGTCGTCCAGATTGTCCGCAACTACAACGCCTACCTTCGGGCCAG CGACGATCTGCCTAAGATGTTCATCGAGTCCGACCCTGGGTTCTTTTCCAACGCT ATTGTCGAGGGAGCTAAGAAGTTCCCTAACACCGAGTTCGTGAAGGTGAAGGG CCTCCACTTCAGCCAGGAGGACGCTCCAGATGAAATGGGTAAGTACATCAAGA GCTTCGTGGAGCGCGTGCTGAAGAACGAGCAGTAA
eGFP	ATGGTGAGCAAGGGCGAGGAGCTGTTCACCGGGGTGGTGCCCATCCTGGTCGA GCTGGACGGCGACGTAAACGGCCACAAGTTCAGCGTGTCCGGCGAGGGCGAGG GCGATGCCACCTACGGCAAGCTGACCCTGAAGTTCATCTGCACCACCGGCAAGC TGCCCGTGCCCTGGCCCACCCTCGTGACCACCCTGACCTACGGCGTGCAGTGCT TCAGCCGCTACCCCGACCACATGAAGCAGCACGACTTCTTCAAGTCCGCCATGC CCGAAGGCTACGTCCAGGAGCGCACCATCTTCTTCAAGGACGACGGCAACTAC AAGACCCGCGCCGAGGTGAAGTTCGAGGGCGACACCCTGGTGAACCGCATCGA GCTGAAGGGCATCGACTTCAAGGAGGACGGCAACATCCTGGGGCACAAGCTGG AGTACAACTACAACAGCCACAACGTCTATATCATGGCCGACAAGCAGAAGAAC GGCATCAAGGTGAACTTCAAGATCCGCCACAACATCGAGGACGGCAGCGTGCA GCTCGCCGACCACTACCAGCAGAACACCCCCATCGGCGACGGCCCCGTGCTGCT GCCCGACAACCACTACCTGAGCACCCAGTCCGCCCTGAGCAAAGACCCCAACG AGAAGCGCGATCACATGGTCCTGCTGGAGTTCGTGACCGCCGCCGGGATCACTC TCGGCATGGACGAGCTGTACAAGTAG
MbG9	GCCGTTTCTGATGTTCCGCGTAAGCTGGAAGTTGTTGCTGCGACCCCGACTAGC CTGCTGATCAGCTGGGATGCTCCTACTATGGTTACGCAGTATTACCGTATCACGT ACGGTGAAACCGGTGGTAACTCCCCGGTTCAGGAGTTCACTGTACCTGGTTCCA AGTCTACTGCTACCATCAGCGGCCTGAAACCGGGTGTTGACTATACCATCACTG TATACGCTGTTACTTGGCGGTGGCGGTATAGCAAGCCAATCTCGATTAACTACC GTACCAGC
Mb035	GCCGTTTCTGATGTTCCGCGTAAGCTGGAAGTTGTTGCTGCGACCCCGACTAGC CTGCTGATCAGCTGGGATGCTCCTACTATGGTTACGCAGTATTACCGTATCACGT ACGGTGAAACCGGTGGTAACTCCCCGGTTCAGGAGTTCACTGTACCTGGTTCCA AGTCTACTGCTACCATCAGCGGCCTGAAACCGGGTGTTGACTATACCATCACTG

	TATACGCTGATTCTTTTGAAGATCCAACTTGTACTTTGGTTACTTCTTCTGGTGCT TTTCAATATATCTCGATTAACTACCGTACCAGC
MbFG EVO	GCCGTTTCTGATGTTCCGCGTAAGCTGGAAGTTGTTGCTGCGACCCCGACTAGC CTGCTGATCAGCTGGGATGCTCCTACTATGGTTACGCAGTATTACCGTATCACGT ACGGTGAAACCGGTGGTAACTCCCCGGTTCAGGAGTTCACTGTACCTGGTTCCA AGTCTACTGCTACCATCAGCGGCCTGAAACCGGGTGTTGACTATACCATCACTG TATACGCTGATTCTTTTGAAGATCCAACTCCGCGTTTGACGCCTTCTCCGGGTGC TTTTCAATATATCTCGATTAACTACCGTACCAGC
PDBody	GCCGTTTCTGATGTTCCGCGTAAGCTGGAAGTTGTTGCTGCGACCCCGACTAGC CTGCTGATCAGCTGGGATGCTCCTACTGCGCGTGTTACGTATTACCGTATCACGT ACGGTGAAACCGGTGGTAACTCCCCGGTTCAGGAGTTCACTGTACCTGGTTCCA AGTCTACTGCTACCATCAGCGGCCTGAAACCGGGTGTTGACTATACCATCACTG TATACGCTGATTCTTTTGAAGATCCAACTCCGCGTTTGACGCCTTCTCCGGGTGC TTTTCAATATATCTCGATTAACTACCGTACCAGC
MonobodyFG LoopLibrary	GCCGTTTCTGATGTTCCGCGTAAGCTGGAAGTTGTTGCTGCGACCCCGACTAGC CTGCTGATCAGCTGGGATGCTCCTACTATGGTTACGCAGTATTACCGTATCACGT ACGGTGAAACCGGTGGTAACTCCCCGGTTCAGGAGTTCACTGTACCTGGTTCCA AGTCTACTGCTACCATCAGCGGCCTGAAACCGGGTGTTGACTATACCATCACTG TATACGCTGATTCTTTTGAAGATCCAACTNNKNNKTTGNNKNNKTCTNNKGGTG CTTTTCAACATGGTTCGATTAACTACCGTACCAGC
MonobodyBC LoopLibrary	GCCGTTTCTGATGTTCCGCGTAAGCTGGAAGTTGTTGCTGCGACCCCGACTAGC CTGCTGATCAGCTGGGATGCTCCTNNKNNKNNKNNKNNKTATTACCGTATCACG TACGGTGAAACCGGTGGTAACTCCCCGGTTCAGGAGTTCACTGTACCTGGTTCC AAGTCTACTGCTACCATCAGCGGCCTGAAACCGGGTGTTGACTATACCATCACT GTATACGCTGATTCTTTTGAAGATCCAACTCCGCGTTTGACGCCTTCTCCGGGTG CTTTTCAATATATCTCGATTAACTACCGTACCAGC
